# A Review of the Methods of Modeling Multi-Phase Flows within Different Microchannels Shapes and Their Applications

**DOI:** 10.3390/mi12091113

**Published:** 2021-09-16

**Authors:** Awatef Abidi, Amir Ahmadi, Mojtaba Enayati, S. Mohammad Sajadi, Hooman Yarmand, Arslan Ahmed, Goshtasp Cheraghian

**Affiliations:** 1 Physics Department, College of Sciences Abha, King Khalid University, Abha 61421, Saudi Arabia; amabedei@kku.edu.sa; 2 Research Laboratory of Metrology and Energy Systems, Energy Engineering Department, National Engineering School, Monastir University, Monastir 5000, Tunisia; 3 Higher School of Sciences and Technology of Hammam Sousse, Sousse University, Hammam Souse 4011, Tunisia; 4 Department of Mechanical Engineering, Arak Branch, Islamic Azad University, Arak 38361-1-9131, Iran; amir.ahmadi7192@yahoo.com (A.A.); menayati@troy.edu (M.E.); 5 Department of Nutrition, Cihan University-Erbil, Kurdistan Region, Erbil 44001, Iraq; smohammad.sajadi@gmail.com; 6 Department of Phytochemistry, SRC, Soran University, KRG, Soran 44008, Iraq; 7 Department of Mechanical Engineering, Faculty of Engineering, University of Malaya, Kuala Lumpur 50603, Malaysia; hooman_yarmand@um.edu.my; 8 Department of Sustainable Design Engineering, Faculty of Industrial Design Engineering, Delft University of Technology, 2628 CE Delft, The Netherlands; 9 Department of Mechanical Engineering, Sahiwal Campus, COMSATS University Islamabad, Islamabad 57000, Pakistan; arslanahmad@cuisahiwal.edu.pk; 10 Independent Researcher, 38106 Braunschweig, Germany

**Keywords:** multi-phase flows, microchannels, micropumps, microturbines, micromixers

## Abstract

In industrial processes, the microtechnology concept refers to the operation of small devices that integrate the elements of operational and reaction units to save energy and space. The advancement of knowledge in the field of microfluidics has resulted in fabricating devices with different applications in micro and nanoscales. Micro- and nano-devices can provide energy-efficient systems due to their high thermal performance. Fluid flow in microchannels and microstructures has been widely considered by researchers in the last two decades. In this paper, a review study on fluid flow within microstructures is performed. The present study aims to present the results obtained in previous studies on this type of system. First, different types of flows in microchannels are examined. The present article will then review previous articles and present a general summary in each section. Then, the multi-phase flows inside the microchannels are discussed, and the flows inside the micropumps, microturbines, and micromixers are evaluated. According to the literature review, it is found that the use of microstructures enhances energy efficiency. The results of previous investigations revealed that the use of nanofluids as a working fluid in microstructures improves energy efficiency. Previous studies have demonstrated special attention to the design aspects of microchannels and micro-devices compared to other design strategies to improve their performance. Finally, general concluding remarks are presented, and the existing challenges in the use of these devices and suggestions for future investigations are presented.

## 1. Introduction to Microstructures

**Figure 1:**
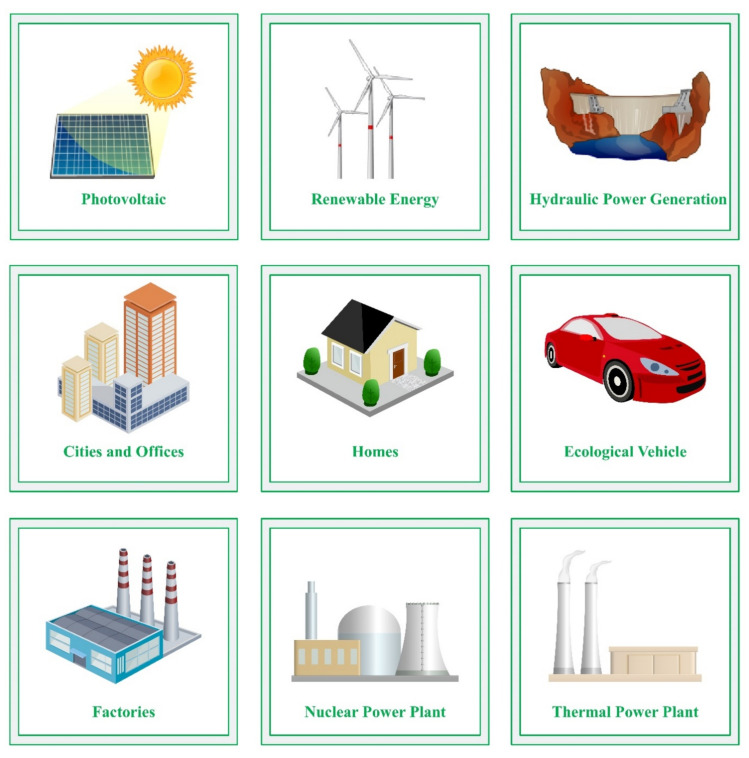
Different applications of MCHs.

Understanding of fluid flow at micro/nanoscale, followed by the development of technologies needed in this field, result in the emergence of a new branch in fluid mechanics that deals with the fabrication of micro-/nano-devices. The main components of micro-/nano-devices are microchannels, where the fluid flow in them requires the existence of a pressure gradient. Microchannels are found in a wide range of devices that handle single-phase and multi-phase fluid flow. Primary applications included micromachine devices, including micropumps, microvalves, and microsensors [1,2]. This subject has been addressed with advances in biology and life sciences due to the need for biological materials such as proteins, DNA, cells, embryos, and chemicals. Micro-device manufacturing has given a suitable foundation for scientific study in microfluidic systems and micro-electro-mechanics (Figure 1). These systems are used in a variety of technical disciplines, including biological treatments, chemical analysis, and electronic device cooling. Although the study of micro-devices involves issues and phenomena that are not important at the macroscale, the dimensions of the system are still sufficient to assume that flow continuity is acceptable in most cases [3,4,5,6]. Working Reynolds number in microscale is usually much smaller than one, indicating the importance of viscous forces. This demonstrates a large amount of pressure drop in the flow and eliminates the possibility of instability to mix in the system. The methods to overcome these issues are one of the microfluidic fields. In nanofluid systems, the volume forces are not important, similar to microsystems, and intermolecular forces should be considered [7,8,9,10,11,12]. A fundamental difference in the study of fluid transport in nanoscale with macroscale is that the assumption of fluid coherence is challenged [13,14,15,16]. Therefore, it is no longer possible to use the relevant governing equations of the fluid, such as Navier–Stokes equations, assuming continuity. To better understand fluid movement, suitable simulation approaches such as molecular dynamics and kinetic Monte Carlo methods should be utilized. These methods require appropriate computational capabilities. Today, with the advancement of science in micro and nanoscale, two-phase microflows have become the most important part of the field of micro/nanofluids [17,18,19,20,21,22]. Bubbles and droplets and complex emulsions have a wide range of applications in the fields of drug delivery, tissue engineering, and oil extraction. Today, CFD simulation plays a prominent role in modeling [23,24,25,26,27,28,29,30,31,32,33,34,35]. Due to the importance of understanding the multi-phase process in different industries that use microstructures, the need for the correct prediction of flow regimes and reducing the risks of phase changes, modeling multi-phase flows, and understanding their performance in micro-devices is very important. Prediction of the properties of different phases, such as temperature and pressure, as well as identification of the variations of the interface between different phases, lead to an improvement in studying and analyzing multi-phase systems in microstructures [36,37,38,39,40]. The simulation of the microchannel will be very important for designing bio microfluidic devices dealing with rheological complex fluids such as lubricating greases, blood, saliva, or mucus. As mentioned, simulation of multi-phase flow in micro-devices using computational fluid dynamics is needed to use models in which the volume fraction of the fluid is accurately modeled [41,42]. Hence, modeling the interface between multi-phase, especially in very sensitive situations, such as detecting droplet motion and the governing regimes, is very complicated. The volume of fluid (VOF), the Eulerian–Lagrangian (EL), and the Eulerian–Eulerian (EE) techniques are the three approaches for modeling multi-phase flows (EE). In the VOF method, which is used to analyze multi-phase systems involving two or more immiscible fluids, the main purpose is to know the position of the interface [43,44,45,46]. The EL one is used for two-phase flows with a volume fraction less than 10% of the dispersed phase, in which the dispersed phase is modeled using the Lagrangian point of view and the continuous phase is modeled by the Eulerian point of view. The EE method is suitable for problems where the dispersed phase volume fraction is more than 10%. In this method, both discrete and continuous phases are modeled by the Eulerian approach [47,48,49,50,51,52]. Table 1 shows several investigations of multi-phase flows within micro-equipment. As may be observed, numerical studies have received greater attention from academics in recent years.

According to a review of past studies, the study of fluid flow in the micro/nanoscale is critical, especially in light of human demands for small-scale manufacture of diverse devices. The study of fluid mechanics at the micro/nanoscale is required because the behavior of flow at these dimensions has unique characteristics. Therefore, in recent years, attention to this field of study has increased and a lot of investigations have been done. Thus, this study aims to review previous studies that considered fluid flow in microstructures to provide a comprehensive source for future studies by presenting a summary of previous articles to help new readers. Therefore, first, an introduction to microfluidics is presented. Then, different methods are described, and finally, the results will be analyzed. At the end of the article, the challenges in this field are presented, and suggestions for future studies are given. As can be seen in Figure 1, micro-devices have many applications. They can be used as a potential system in the transportation industry, industrial cooling, nuclear reactors, extraction of energy from thermal and other energy sources, electronic cooling, military fields, space applications, and medical applications [62,63].

### 1.1. Different Flow Regimes

**Figure 2:**
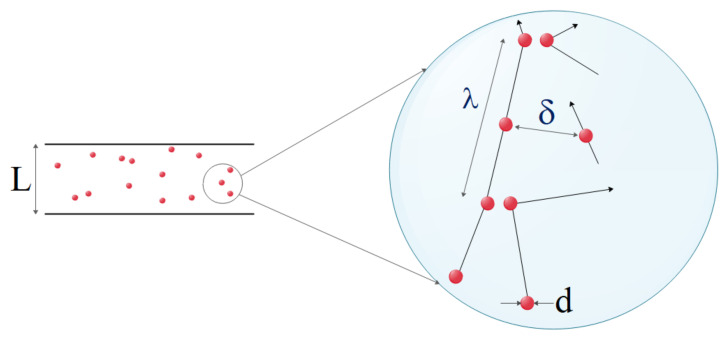
Effective parameters in molecular level modeling [75,76,77,78,79,80,81].

The gas slides with a specified tangential velocity on microchannel walls while passing through. Moreover, a temperature jump between the fluid and the wall is preserved. Another important phenomenon in dilute systems, such as dilute gas flows, exposes when the channel size becomes as small as wherein the molecular interactions control the physics of flow dynamics. The Knudsen number, which is defined as the ratio of the average path of the free gas scan to the system characteristic length, is a novel metric that measures the degree of dilution of the system. The Knudsen number is used to calculate a system’s dilution. The Knudsen number is, in fact, a crucial characteristic of nanoscale and microscale flows. The flow regime in macro-channels is defined by the values of velocity and viscosity, which are represented in the Reynolds number, and compressibility, which is reflected in the Mach number. The flow regime in MCHs is determined by the most significant parameter, dilution, which is defined by the Knudsen number. Although one of the assumptions in equations of Navier–Stokes is continuity, the researchers have proposed that Navier–Stokes is already applicable to MCHs flows where dilution effects become important by using slip boundary conditions. For gas flows that are examined at the micro and nano dimensions, it is necessary to define new parameters for modeling the molecular interactions. At the molecular level, factors, such as the mean of free path (λ), molecular diameter (d), intermolecular distance (δ), must be considered (Figure 2) [64,65,66,67,68,69,70,71,72,73,74].

Another effective parameter is the dimensionless parameter, the Knudsen number, which is used for problems with very small characteristic length or when using very dilute gases. This number is used as an index to validate the continuity equation. The Knudsen number is calculated as follows:

(1) $$kn=\frac{\lambda }{L}$$

where L is the system characteristic length, which might be the hydraulic diameter or channel height. λ is also the mean free path which is defined by [82]:

(2) λ=μρ2RTπ

where μ is the dynamic viscosity, ρ is the gas density, T is temperature, and R is the universal gas constant equal to 287.

**Figure 3:**
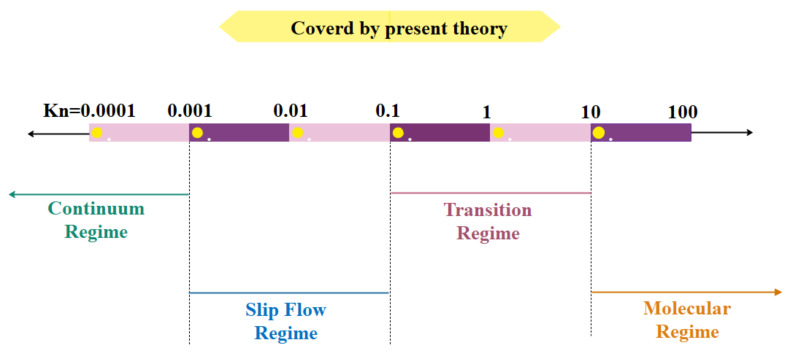
Flow regimes based on Knudsen number [85].

Based on the Knudsen number, flow regimes are divided into four categories, each with its own set of flow characteristics and governing equations. The Knudson number (Kn) must be smaller than one to achieve thermodynamic equilibrium. In micro/nanoscale gas fluxes, the Knudsen number is critical. The Knudsen number is not the sole parameter to consider in restricted flows in micro/nano channels; therefore, the parallel between low-pressure and confined flows is incomplete. Different regimes can, however, be expressed as a function of the Knudsen number [83]. This classification can be stated as follows: in the range of $Kn<10^{-3}$, the continuum assumption is used. Such flows can be modeled with acceptable accuracy by the Navier–Stokes equation with conventional no-slip boundary conditions. In the interval of $10^{-3}<Kn<10^{-1}$, the flow regime is classified in the slip flow regime. The Navier–Stokes equations are still valid except that slip velocity and thermal jump must be applied at the walls. These new boundary conditions indicate that the dilution effect is first observed on the wall. In Knudsen numbers of $10^{-1}<Kn<10$, the flow is considered as a transient flow and the continuity assumption and Navier–Stokes equations break down. Molecular collisions still need to be addressed and cannot be ignored. Molecules freely flow for Kn numbers larger than 10 and molecular collisions are negligible compared to those collisions with walls [84]. A classification of flow regime, based on Kn number, is depictured in Figure 3 [85].

**Figure 4:**
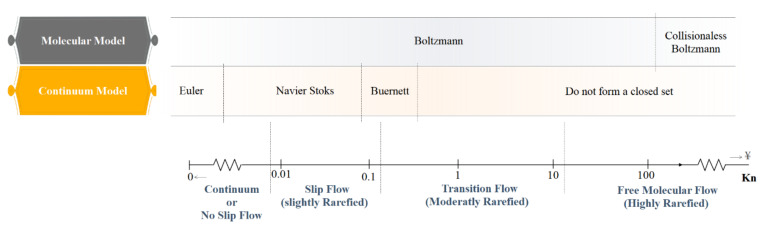
Equations and boundary conditions based on Knudsen number [85].

Figure 4 shows the equations and boundary conditions of each class.

**Figure 5:**
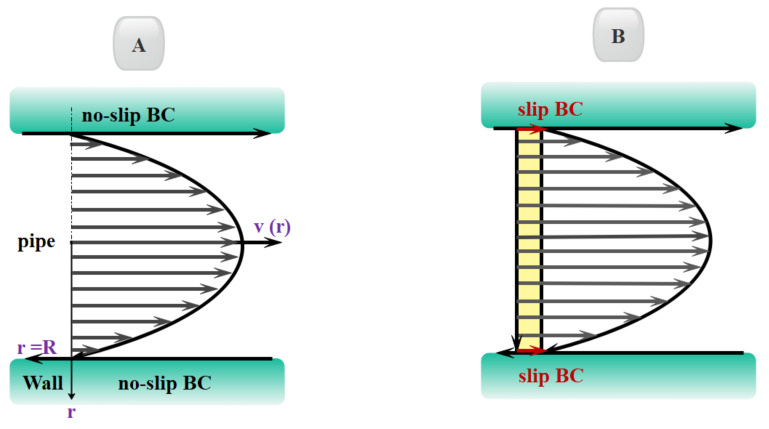
Velocity profile (**A**) in the absence and (**B**) in the presence of slip flow at walls [85].

Microchannels are efficient heat exchangers with lots of advantages, such as high aspect ratio, high convection heat transfer coefficient, low fluid volume required, and small weight and size. The efficacy of such novel tools has been confirmed by researchers and engineers. The slip flow regime governs in most microfluidic devices. As shown in Figure 5, the no-slip boundary condition does not apply to this type of flow and the fluid velocity adjacent to the wall is not equal to the solid wall. For gas flows on the solid surface, there is a Knudsen layer near the solid surface, which has a thickness in the order of λ, and this layer is not negligible in microflows with large Kn numbers [86,87,88,89,90,91,92,93,94,95].

**Figure 6:**
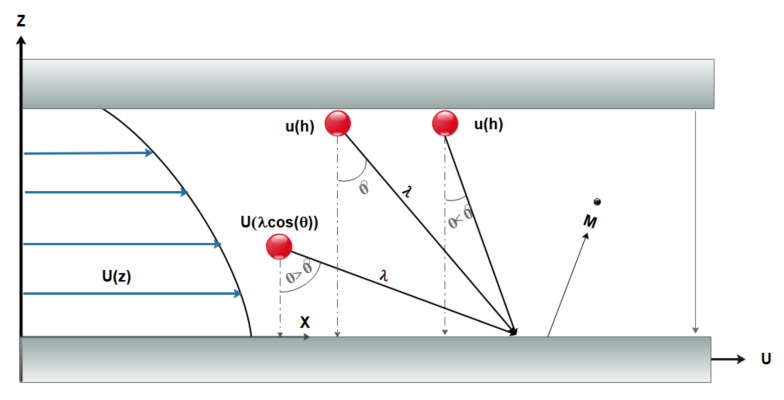
Setup of a system for rarefied gas flow in a channel (Lin Wu [100]).

**Figure 7:**
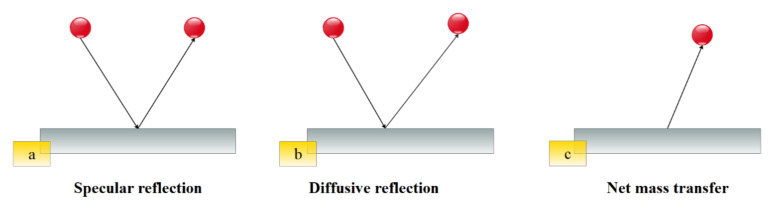
Wall-gas molecule interactions include (**a**) specular reflection of impinging gas molecules, (**b**) diffusive reflection of impinging gas molecules, and (**c**) net mass transfer at the wall owing to evaporation/condensation or wall porosity (Lin Wu [100]).

In the slip flow regime, the Navier–Stokes and energy equations are still applicable in the bulk of the channel [96,97], and only in the vicinity of the channel known as the Knudsen boundary layer is needed to define new boundary conditions. In the slip flow regime, because of the thermodynamic imbalance of flow on the wall, Navier–Stokes equations with a no-slip boundary condition cannot be used and it is necessary to use new modeling to study fluid flow. In order to model the boundary conditions in the slip flow regime, the first and most fundamental description of gas–surface interaction, based on particle collisions and reflections, is provided by Maxwell [98]. In this model, he suggested a correction coefficient for the tangential momentum to adjust the slip velocity and to determine a fraction of gas molecules that is diffused from the solid surface in the microfluid. Many studies have shown that this coefficient is sensitive to the conditions of the gas–solid interface, the gas particles, the material, temperature, and roughness of surface [99]. There are two categories of methods for analyzing fluid flow in these cases. Methods that assume fluid continuity using the Navier–Stokes equations with a slip condition and other methods at a molecular level, such as Molecular Dynamics and Direct Simulation Monte Carlo [100] in which the fluid is a set of separated particles (Figure 6 and Figure 7). The first methods are cost-effective in terms of computational cost. They are also simple and efficient computational methods [99,101].

As tabulated in Table 2, the channels could be classified based on their hydraulic diameter. It is a simple yet efficient way to study various situations. Regarding the available results, it seems that the MCHs are selected based on the slip condition [64].

Mehendale et al. [102] have proposed a classification whereby channels with the diameters of 1 μm to 100 μm are called micro heat exchangers, 100 μm to 1 mm are called meso heat exchangers, 1 mm to 6 mm are called compact heat exchangers, and larger than 6 mm are called conventional heat exchangers. Gas dilution in atmospheric pressure was considered by Kandlikar and Grande [103]. They sorted channels based on their diameter from smallest to powerful in Table 3

One of the characteristics of biological flows, which move through narrow channels, is low Reynolds number regime, and different modeling approaches are needed to solve such problems. Electrokinetic forces play an important role in these systems, while, for example, lots of questions about a two-phase flow in a channel of less than 10 μm wide remain unanswered. Therefore, the initial definition presented by Kandlikar and Grande [103] for channel classification has undergone some changes. They have provided a general definition based on the smallest channel dimension, the results of which are indicated in Table 3.

D_h_ is the diameter, for circular cross-section, or the smallest characteristic length, for non-circular cross-section channels (Table 2). For instance, Dh is the small dimension of a rectangular cross-section. This presented classification of channels is the simplest one. In reality, whether the continuum assumption is valid or not for the gaseous flow, it is not easily recognizable and needs a careful check for each case.

### 1.2. Intra-Microchannel Flow Modeling Methods

**Figure 8:**
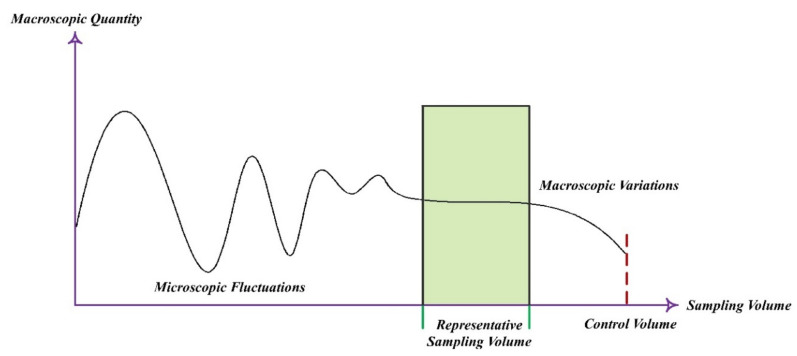
An example of a control volume (shaded area) where a continuum assumption is established.

In general, fluid flow problems can be solved in either Lagrangian or Eulerian. In the latter approach, the medium is considered continuous and consequently, the continuity and Navier–Stokes equations are governed. In this case, the computational domain is comprised lots of control volumes wherein the thermophysical and mechanical properties are constant in every single volume and vary from control volume to control volume. As a result, in any control volume, the continuity and Navier–Stokes equations are true. Because each control container contains a high number of molecules, the entrance and exit of molecules have no effect on the mechanical and thermodynamic characteristics of the fluid in each control volume. In other words, when the continuity assumption is preserved, microscopic or molecular oscillations should not be more important than averaged values properties. Therefore, the control volume should be large enough to ignore the microscopic fluctuations and on the other hand, it should be small enough to capture macroscopic changes (Figure 8). In the control volume shown in Figure 8, the fluid can be considered continuous because of the negligible molecular fluctuations [104,105,106,107,108,109,110,111,112,113,114].

In the Lagrangian approach, due to the high molecular oscillations of the fluid, the computational domain cannot be considered continuous. In this case, each molecule has its own mechanical and thermodynamic properties. Thus, molecules have to be solved separately. Obviously, solving equations in the Eulerian approach is much simpler than the Lagrangian. For instance, imagine airflow within a channel with the velocity of 0–1 m/s, the fluid flows almost in the same direction as the channel axis, although the fluid flows in any direction with molecular velocities in the order of 1 km/s [115,116,117,118,119,120].

**Figure 9:**
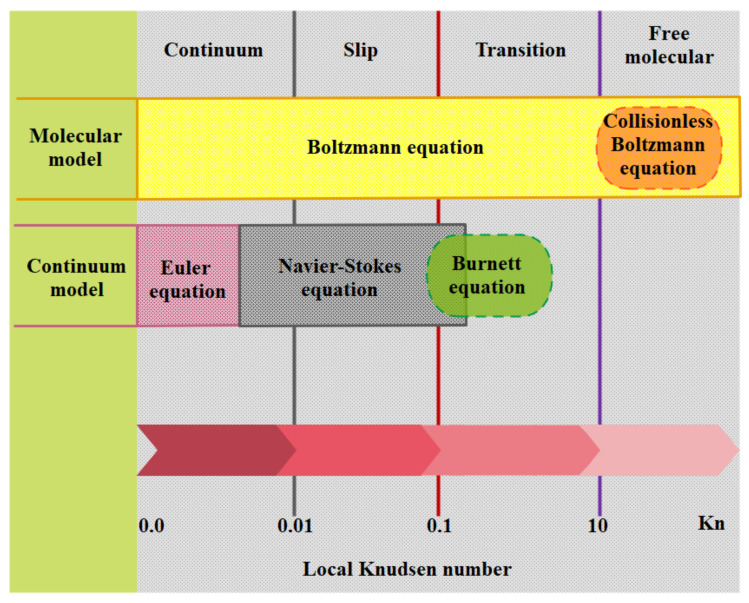
Modeling approaches and correlations of different flow regimes.

For each control volume comprising thousands of molecules, a set of continuity, momentum, and energy equations will be employed. Every single molecule requires the solution of the equations of continuity, momentum, and energy at the microscopic level. With the increasing of the number of equations, computational cost increases so that using CFD methods requires high technology and powerful supercomputers. As shown in Figure 9, although new methods such as LBM have emerged to solve flows at the microscopic level, many disadvantages are still observable [120,121,122,123,124,125,126].

### 1.3. Study of Slip Velocity Models and Lattice Boltzmann Method

**Figure 10:**
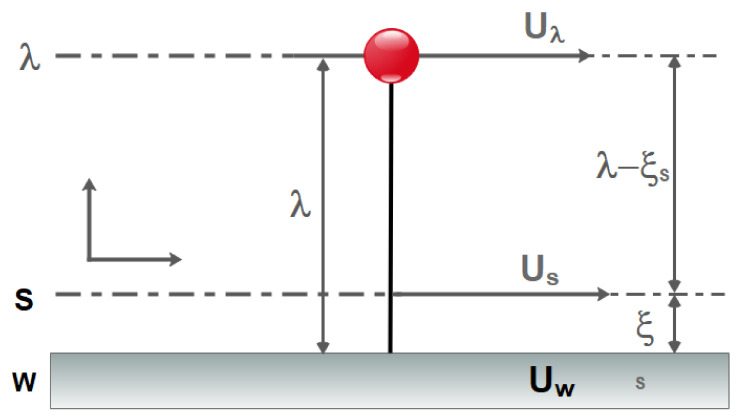
A schematic view of the fluid flow at the solid surface. λ represents the fluid layer one mean-free path away from the solid surface *w*. Surface *s* is the slip surface $\xi_{s}$ away from the solid wall and $\lambda -\xi_{s}$ away from the surface λ [132].

Many researchers developed the first slipping velocity model which was proposed by Maxwell. Myong et al. [127] presented a model that takes the effect of surface curvature into account. Dongari et al. [128] solved the Navier–Stokes equations through the use of a second-order slip boundary condition and compared the results with the Boltzmann equation. It was found that the aforementioned boundary condition is valid for Knudsen number up to 5. Shen and Chen [129] showed that the Boltzmann equation is the first-order dependent on the Knudsen number. Therefore, second-order and 1.5-order models are merely mathematical expansions that do not match the nature of the flow. They developed a new model derived from the Boltzmann equation. The first-order slip velocity correlation is computed using the following Kundt and Warburg theory [130].

(3) $$U_{slip}=U_{s}-U_{wall}=\zeta {\left(\frac{\partial U_{s}}{\partial U_{wall}}\right)}_{n}$$

where *n* is the direction normal to the wall and ζ is the slip coefficient. Wu [131] proposed a new model using the gas kinetic theory and improved the Reynolds lubrication equation using the aforementioned new boundary condition. He claimed that this equation is applicable to air at any Knudsen numbers. According to Figure 10, Zhang [132] presented a second-order model and stated that this model is suitable for micro/nanoflows.

Assuming the spherical and hardness of the particles and using the Maxwell boundary condition for the wall-colliding particles, Gibelli [133] proposed a new slip boundary condition based on gas kinetic theory. Szalmás [134] described a method for calculating the viscous velocity, diffusion, and thermal slip factors of three-component gaseous mixtures. At increasing rarefaction values, it was discovered that the slip flow approximation offers a pretty reasonable estimate of flow rates.

Colin [135,136] reviewed a variety of analytical and numerical methods for studying convection in microchannels. He has investigated the impacts of dilution under constant heat flow and constant temperature boundary conditions, as well as the relevance of the conduction term and viscous dissipation effect in various solution techniques. It was found that more and more experiments are needed to develop research in this area. Using LBM, Sbragaglia and Succi [137] examined different boundary conditions. 

They discovered that by employing second-order accuracy in terms of the Knudsen number, the slip coefficient in the wall could be modified to obtain a satisfactory match with the analytical and experimental data by Qi et al. [138], who investigated the influence of surface wettability on wall sliding. It was discovered that the material of the microchannel has a significant impact on surface wettability, implying that material selection is a key element in altering slip length. In a two-dimensional microchannel, Nikkhah et al. [139] studied the forced convective heat transfer of water/functionalized multi-walled carbon nanotube (FMWCNT) nanofluid. The local Nusselt number varies in a periodic way over the length of the microchannel and rises with the growth in the Reynold number, according to the findings. Nguyen et al. [140] analyzed the impacts of the magnetic field in a slippery microchannel. Applying a magnetic field to the microchannel enhanced both slip velocity and temperature gradient close to the wall, according to the findings.

Niu et al. [141] studied the non-Newtonian sliding flow of Water-Al2O3 in two-dimensional microtubes. They discovered that increasing the volume percentage of solid nanoparticles and the duration of the slide had a substantial impact on nanofluid behavior. Bahrami et al. [142] created a microchannel with an injection wall that was set to slip condition. Furthermore, the injections were delivered through ribs into the microchannel. The findings of the research indicated that the height of the rib induces a higher heat transfer and slip velocity near the wall. Cercignani and Daneri [143] solved the linear Boltzmann equation under isothermal conditions and a modest pressure gradient. The interaction of the wall and the gas was described using Maxwell’s scattering concept. Xue and Fan [144] substituted the hyperbolic tangent function of the Knudsen number for the Knudsen number in the slip term. They also compared the model’s predictability to Direct Simulation Monte Carlo (DSMC) estimates. Pan et al. [145] examined the effect of wall temperature, wall velocity, mass, diameter, and number density of gas molecules on the slip coefficient for five gases using the DSMC technique. They discovered that the slip coefficient was unaffected by any of the other variables. Karniadakis and Beskok [146] solved the flow velocity and pressure distribution using a higher-order slip model without accounting for inertial components. On the differential version of Navier–Stokes equations, Zohar et al. [147] used the perturbation approach. They solved the problem with a Knudson number of 0.1 and ignored the slip boundary condition’s second-order terms. El-Genk and Yang [148] and Celata et al. [149] examined the water flow dynamics in a microtube with the slip boundary condition in an experimental investigation using the test loop. According to their findings, the slide length is between 0.7 μm to 1 μm.

**Figure 11:**
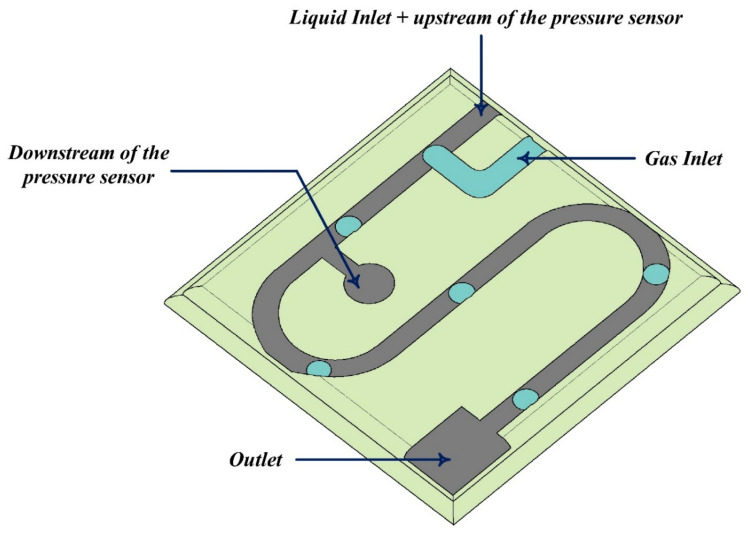
In this experiment, a microchannel was employed. The entire plate is 30 mm × 60 mm × 6 mm. The T-junction is in the upper-left corner, and an auxiliary channel is bored 30 mm downstream to plug the sensor’s low-pressure side. [161].

In recent years, the use of grid-based Boltzmann methods for simulating flow in micro-dimensional geometries has been the interest of researchers. Surface effects at the microscale have much more dramatic effects than metric scales. For example, the no-slip boundary condition, which is widely used on a metric scale, is no longer valid in microchannels, and the slip flow should be taken into account. At this scale, other methods such as LBM or molecular dynamics should be used [150,151,152,153,154,155]. Although direct simulation methods such as Monte Carlo or Lattice Boltzmann can be used for flows with Kn>0.1, researchers should compensate between the high computational cost of such methods and their unique abilities [125]. In recent years, the LBM has been considered as a powerful numerical method for simulating microscale fluid flow. Previous studies [156,157,158] have demonstrated that the LBM has the capability of simulating the large Knudsen number flow regime with high accuracy in complex geometries. The main reason for using these methods is their capability of simulating microscale flows without the need for satisfying continuity equation. The thermal LBM was utilized by Wang and Yang [159] to investigate the effects of the Knudsen number on heat transfer and flow dynamics in a microchannel. They looked at the thermal leap and slip velocity at various Knudsen numbers, as well as the temperature distribution in a microchannel. Tian et al. [160] studied gas flow and heat transfer in a microchannel using thermal LBM with viscous dissipation and suggested novel boundary conditions for slip velocity and a thermal leap based on macro characteristics. To mimic two-phase flows in microchannels, Riaud et al. [161] created a new LBM dubbed color-field LBM. They compared their numerical results to those of the experiments. This technique, as illustrated in Figure 11, has a high level of agreement.

Alizadeh et al. [162] used LBM to investigate the influence of temperature on ion distribution in an electro-osmotic flow inside a planar microchannel on the walls and presented a novel model. Lin and Chen [163] investigated the influence of surface potential, ion concentration, channel height, and periodic electric and pressure fields on an electro-osmotic flow with heterogeneous surface potential in a microchannel using the Poisson–Boltzmann technique.

**Figure 12:**
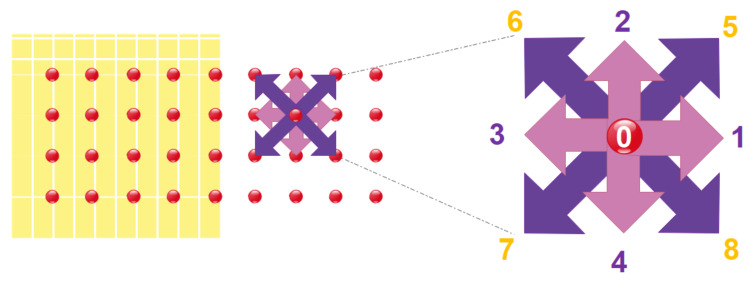
A D2Q9 model arrangement investigated by Shirani and Jafari [166].

Their findings indicate that vortices in the center and near the wall may be generated in a microchannel using heterogeneous surface potentials and stimuli with various phase angles. Yang and Lai [121] studied the heat transfer of a low-Reynolds 47 nm water alumina nanofluid flow in a microchannel with constant-temperature walls and a rectangular heat sink using a computer model. They discovered that when particle volume and Reynolds number grew, so did the Nusselt number. Nie et al. [164] developed the conventional LBM by proposing a local density dependency for the under relaxation factor to simulate the flow in micro-dimensional geometries. In the calculation of the under-relaxation factor, a parameter was used, which originated from the experimental results. They used their method to study slip velocity and non-linear pressure drop along microchannels. Tang et al. [165] modified the method proposed by Nei et al. [164] to be independent of experimental results by considering a gas flow in a microchannel. To take slip velocity into account, they presented a bounce-back boundary condition. They compared their results, the distributions of velocity and pressure throughout the microchannel, with that of other researchers. A similar boundary condition was used by Shirani and Jafari [166] to simulate microflows using the D2Q9 model shown in Figure 12. The comparison between their results and those of analytical and experimental presented in the literature was showed a good agreement.

**Figure 13:**
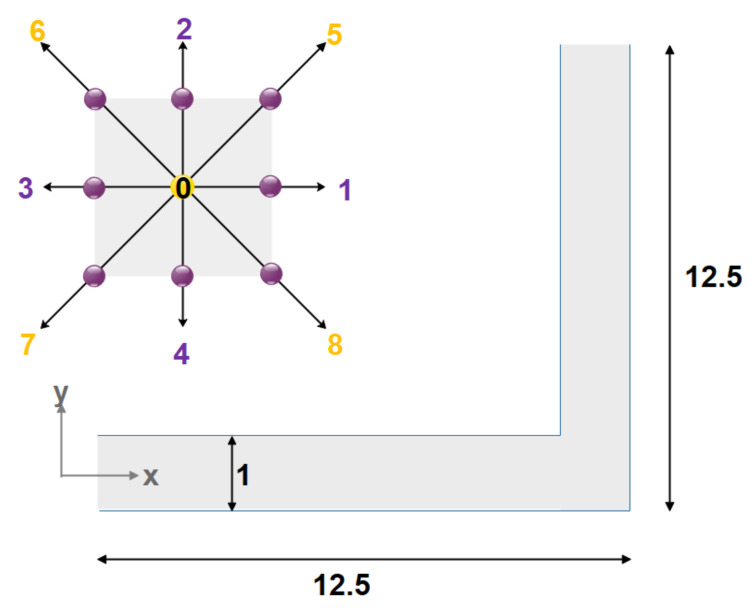
Domain of computation. Computational nodes. There are eight moving particles (numbered 1–8) and one stationary particle (0) .The arrow that corresponds to a particle’s motion indicates its direction of motion. [179].

To study the effect of Knudsen number, Perumal et al. [167,168] applied LBM with a singular under relaxation factor on a uniform mesh to simulate flows within a micro-device. A simulation for the microflows with LBM was conducted by Prasianakis and Ansumali [169]. They simulated the Couette flow at different Knudsen numbers and compared the results against the analytical results of the literature. Wang et al. [170] conducted a review on gas microflows in LBM. They have discussed on various boundary conditions and the proposed models for the relaxation time, along with their advantages and disadvantages. Lee and Lin [171] released more accurate results of slipping flow by proposing a novel model for the Knudson number and using second-order accuracy terms. Using LBM, Wang et al. [172] investigated the mixing of electro-osmotic flow within a plane microchannel in the presence of square obstacles. Poisson–Boltzmann, Navier–Stokes, and mass transfer equations were solved. Boundary conditions in the LBM are very delicate and have a great impact on the convergence of the solution. Zhao-Li et al. [173] have used first-order non-equilibrium extrapolation for velocity and pressure at the boundaries to calculate unknown nodes. This method has higher numerical stability than the method of Chen et al. [174]. The first-order Maxwell slip boundary condition was used by Reis and Dellar [175] to simulate dilute multi-phase flows in microchannels. This method has second-order accuracy for compressible flows. According to Jeong’s study [176], the deviations reported from different studies in dilute gas flows are originated from different boundary conditions and the presentation of various correlations between Knudsen number and relaxation time. D’Orazio et al. [177] used LBM to investigate steady-state forced convection of a water–Cu nanofluid via a microchannel at three Reynolds numbers: 1, 10, and 50, as well as three slip coefficients: 0.001, 0.01, and 0.1. Under slip flow circumstances, the microchannel was partially exposed to continuous heat flux and partly insulated without considering temperature rise. The influence of nanoparticle diameter on nanofluid viscosity was ignored in this study. They discovered that raising the solid volume percentage and slip coefficient increases the average Nusselt number at any fixed Reynolds number. At higher Reynolds numbers, the rise was more pronounced. Liu and Guo [178] conducted a study of a pressure-driven flow within a lengthy microchannel to investigate the effects of dilution and compressibility on the pressure distribution along the microchannel. The pressure profile’s departure from a linear distribution was investigated. LBM is a numerical approach for fluid flow simulation that solves the discretized Boltzmann equation rather of the Navier–Stokes equations, according to the article. Micro gas flows have a high Knudsen number because of the tiny size of the system. Instead of Navier–Stokes equations, certain techniques based on gas kinetic theory, such as the Direct Simulation Monte Carlo (DSMC) and LBE method, are recommended in discontinuous flow regimes where molecule interactions become significant. The LBE technique has two key characteristics that allow it to model flows with high Kn = 1. The collision of gas molecules with walls is taken into account, as well as the second-order slip boundary condition. In a numerical simulation, Liu and Guo [178] studied the effects of different pressure ratios and Knudsen numbers on the flow pattern; also, the relationship between non-linear pressure distribution and averaged Knudsen number is also investigated. The gas flow dynamics inside a microchannel with a 90-degree bend, illustrated in Figure 13, was simulated by Agrawal et al. [179] for the range of 0.02<Kn<0.06.

Using LBM, Agrawal et al. [179] studied gas flow through a microchannel with a single 90-degree bend without heat transfer. They concluded that the pressure and velocity distributions are quite similar to the straight microchannel, although the flow rotation near the bend exists even when the Reynolds number is less than one. They also concluded that the mass flow rate of the curved microchannel is about 1% up to the straight one.

### 1.4. Compressible Flow within Microchannels

**Figure 14:**
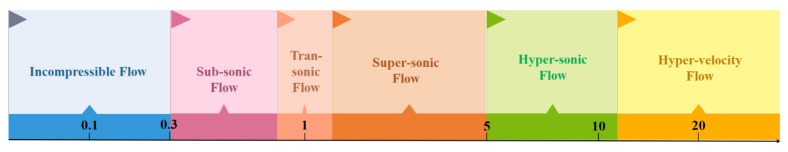
Various flows in different Ma numbers.

The majority of microelectronics are made up of a series of microchannels. Surface-to-volume ratio, slip flow, viscous dissipation, compressibility, dilution, and intermolecular forces are all essential in micro-devices. The flow dynamics characteristics, which are dependent on surface phenomena, have a significant influence on the system’s performance due to the huge surface-to-volume ratio. Fluid compressibility becomes relevant in the case of gas flow within microscale devices when the Mach number exceeds 0.3 [180], as illustrated in Figure 14. Although, for Mach numbers larger than 0.2, it was advised to examine the influence of compressibility on the gas flow [181].

The heat transfer of gas flow in a semi-circular microchannel was numerically studied by Languri and Hooman [182]. By considering slip boundary conditions and temperature jumps, they showed that unlike a fully developed flow, the Nu number varies with Re and Pr numbers through the developing zone. Using the DSMC method, White et al. [183] investigated a dilute argon gas flow through a straight microchannel and compared the results against a single 90°-bend microchannel and, in another microchannel, with a couple of 90° bends in different values of Knudsen number. The distribution of pressure and the variation of Mach number along the straight microchannel are almost the same as the bended ones, except through corner regions. The deviations along bend zones are due to fluctuations in pressure and the Mach number there. It was approved that bending microchannel results in an increased mass flow rate for the range of 0.02<Kn<0.08. In this study, two profiles for the slip velocity and the shear stress in different Knudsen numbers were presented. The maximum increase in mass flow rate can be observed for different values of Knudsen number by increasing the number of bends. The compressible slip flow between the cylinders has been studied by Rohlf [184]. The viscosity was considered density-dependent, and a non-linear second-order differential equation was used to model the density. Pressure and its gradient, which was modeled as a second-order non-linear derivative term, were plotted versus density. Moreover, the velocity at the centerline, which was drawn along the microchannel for large Reynolds numbers, was mathematically modeled as a quartic polynomial function. The results are compared with the results of the numerical solution of Low-Reynolds flows where their viscosity has a linear relationship with density. The results of this study have implications for the study of blood flow. In the constrictions with the smallest Reynolds and Mach numbers, there is fairly good agreement in the mildest and more severe constrictions considered, for both slip and no-slip conditions. It can be observed that the density tends to zero for sudden constrictions. Considering the effect of the slip boundary condition and temperature jump, Sai and Khasaneh [185] investigated semi-isothermal gas flow within a two-dimensional microchannel in a low magnetic Reynolds number and Hartman numbers of less than unity. By studying the pressure drop, magnetic forces, and wall shear stress, they simplified the governing equations of the gas flow dynamics under the influence of the magnetic field [186,187,188,189].

**Figure 15:**
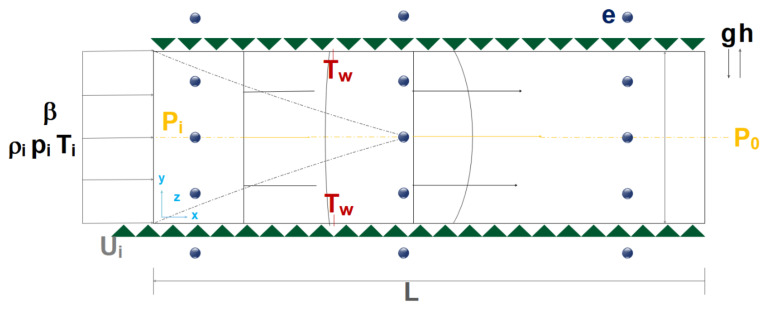
Model of analysis. e = (0, 0,e_0_) and h = (0,h_0_, 0) [190].

The Microchannel shown in Figure 15 was studied by Weng and Chen [190]. The creation of an electromagnetic driving force in ionized gas pressure-driven microflows under the influence of magnetic and electric fields and presented a mathematical model in terms of dimensional numbers. The slip velocity and the flow resistance were found that can be increased by the generated driving force for the range of −10<Ha<10. The effects of electromagnetic driving force and joule heating on velocity slip and temperature jump are found to be amplified as the Knudsen number grows, whereas the effects on flow drag and heat transfer rate are found to be reduced.

Wang et al. [191] investigated the flow pattern of the ideal gas within a microchannel by solving the Navier–Stokes and continuity equations while considering a high-order slip model. This model, it was claimed, could be applied to any Knudsen number, independent of the gas’s bulk velocity. The slip model, on the other hand, has a lot of unknown coefficients. There is another solution acquired from the Boltzmann equations, in addition to the preceding technique, which starts with an analytical solution using the Navier–Stokes equations.

## 2. Multi-Phase Flows within Microchannels

### 2.1. Flow Boiling and Bubble Growth in Microchannels

**Figure 16:**
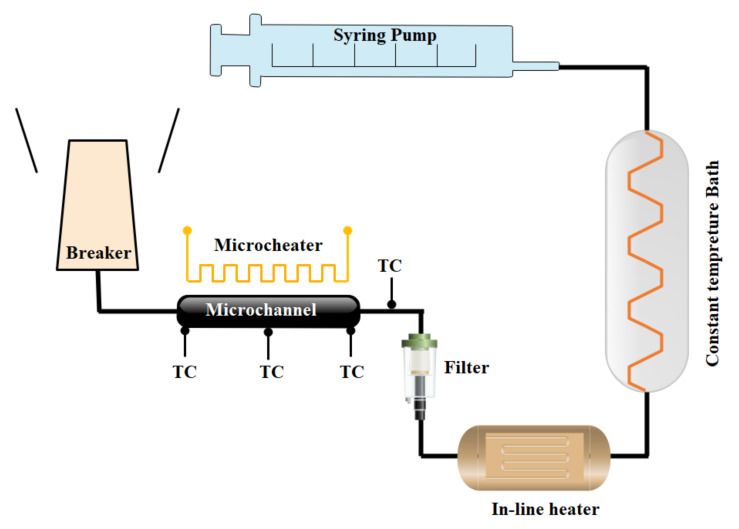
Schematic of experimental microchannel flow loop [197].

Investigating microchannels systems, including flow in capillaries and other micro-sized tissues, computer chips, etc., has attracted much attention in the field. Industrial processes are usually accompanied by heating and boiling [63,192,193,194,195]. In microchannel flows, when the wall temperature exceeds the saturation temperature of the fluid, droplet formation, which is an important issue affecting heat transfer, is inevitable. Compared to microchannels, the bubble formation in flow boiling was less sensitive to the flow properties of macro-channels [64]. Mukherjee and Kandlikar [196], in their research on flow boiling in high-diameter tubes and various microchannel widths, observed that the flow inside the microchannel facilitated the rapid migration of droplets from the wall, resulting in slower growth of droplets compared to the pool boiling in the same heat transfer. They observed that the smaller the channel width, the faster the droplet would grow transversely and fill the microchannel. Consequently, the time required for transverse growth is decreased and it will take shorter time for the droplet to block the channel. Increasing the channel width also results in more alignment of the droplets with the flow direction. As shown in Figure 16, the effect of temperature on droplet growth for the previous study was investigated by Mukherjee et al. [197]. As the microchannel wall temperature increases, the time required to increase the droplet diameter decreases. Moreover, the reduction of nanofluid mass flux increases droplet growth. Under the same conditions, the use of high-density nanoparticles increases the time required for droplet formation and droplet growth compared to other low-density nanoparticles.

**Figure 17:**
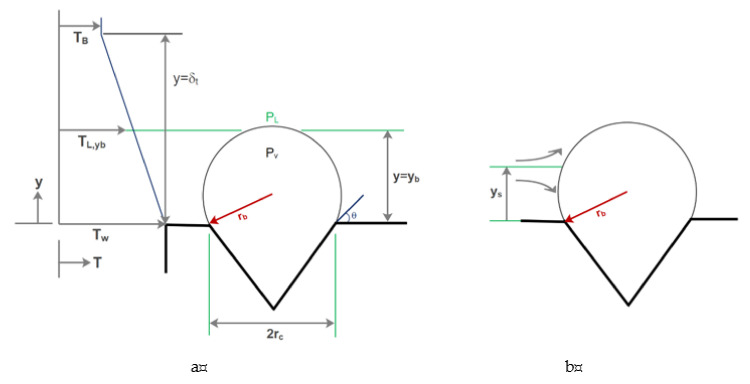
Temperature profile (**a**) and stagnation streamline (**b**) around a nucleating bubble [198].

**Figure 18:**
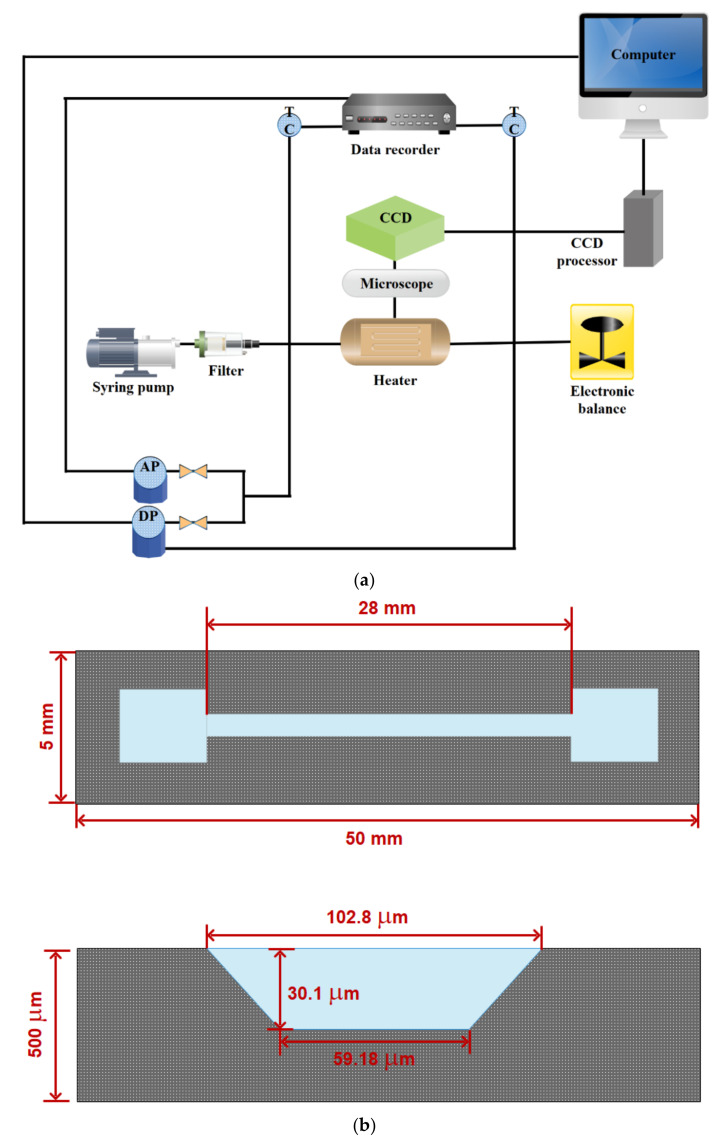
(**a**) Schematic of the experimental setup. (**b**) The top and cross-section views of the test section with a trapezoid microchannel [199].

Kandlikar et al. [198] conducted experiments on the bubble growth process within a brass-made microchannel and showed that in the boiling process, high heat transfer coefficients lead to the formation of the superheat region. Consequently, as shown in Figure 17, it results in the rapid growth of bubbles. As shown in Figure 18, Lee et al. [199], in an experimental study, found that the bubble growth process in the microchannels was isotropic until the bubble diameter was less than the microchannel diameter. It was then pressurized by the walls and significantly affected by the drag force.

**Figure 19:**
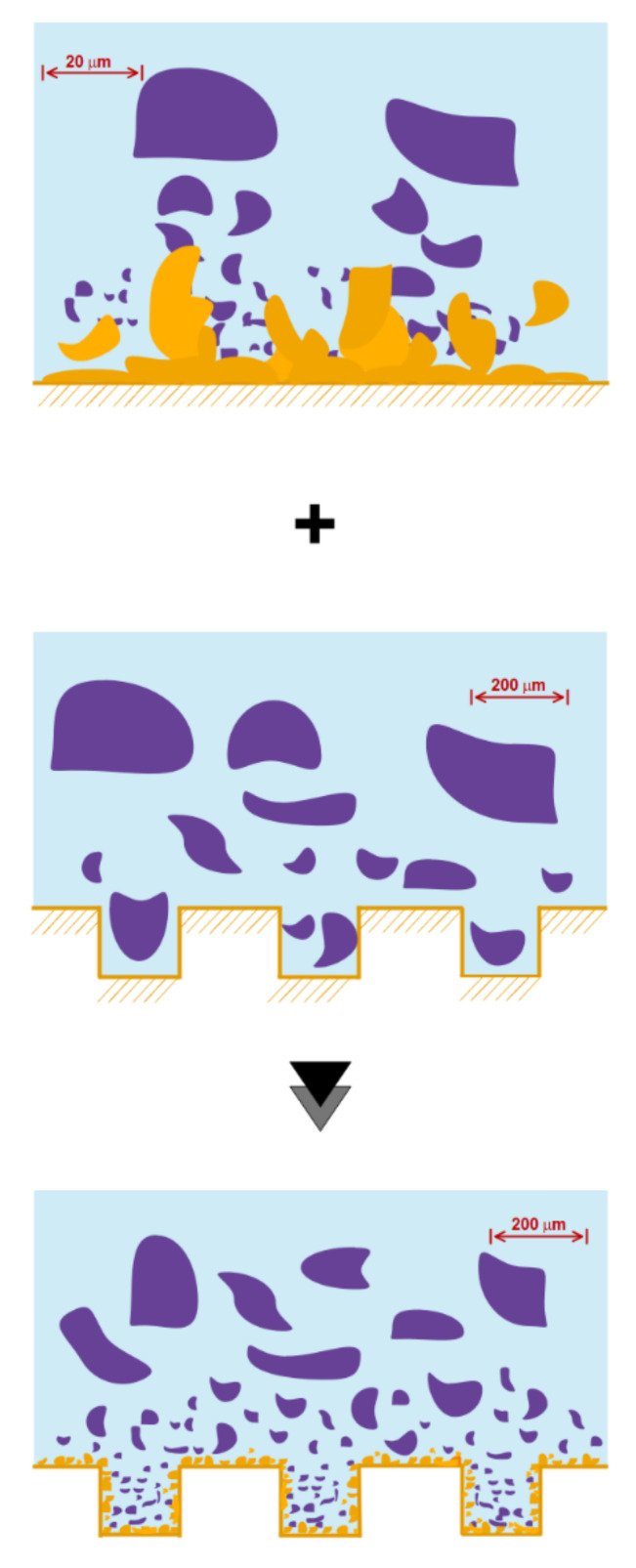
Schematic of bubble dynamics on porous, mesochannel and combination of them in low heat flux [202].

Cornwell and Kew [200] have determined various bubble formation patterns in an experimental study on flow boiling at low-velocity R-111 refrigerants. They conducted experiments on low-width microchannels and concluded that by reducing the microchannel input width, bubbles would grow transversely in the shortest possible time and then begin its longitudinal growth. It was also found that the transverse growth of bubbles occurred in a short length of the microchannel, while most of the microchannel length was attributed to longitudinal growth. Yu et al. [201] simulated gas–liquid flows at small capillary numbers using converged microchannels using LBM. They found that the bubble breakup was exacerbated by the pressure difference in the two insoluble fluids. Gheitaghy et al. [202] studied U-shaped microchannels. They tested the pool boiling in the presence of ionized water by porous surface electrodeposition (Figure 19). In this way, they combined passive surface enhancement techniques with the creation of a fluid vapor pathway and the creation of more nuclear sites. The experimental results showed that the heat flux reached 1650 kW/m^2^ and the convection heat transfer coefficient reached 225 kW/m^2^K. Compared to the smooth surface, the heat flux increases by 100% and HTC shows an increase of about 80%. Young et al. [203] found that bubble growth and its separation from the hot wall are significantly influenced by the thermal boundary layer near the wall. Moreover, bubble growth under the same thermal conditions in a tube is different from the microchannel due to the high shear stress in the flow direction on the wall. It is believed that the behavior of the bubble during its growth stages is controlled by the rate of heat transfer to it.

### 2.2. Bubble Formation in Microchannels

It has been pointed out that in recent years, understanding microscale physical phenomena are of particular importance due to their widespread use in the industry, and especially in the medical sciences. One of the major challenges in studying fluid dynamics at the microscale is to investigate the drop formation process in microchannels to control the size of the bubbles. The bubble formation process of two immiscible fluids in microchannels has many applications, such as food industry and polymer production, DNA analysis, and microreactors [204,205,206,207]. Abrishamkar et al. [208] simulated droplet formation at a flow focusing device with transverse flows using COMSOL software. They investigated the effect of the relative velocity of water (discontinuous phase) and oil (continuous phase) on the bubble size. The simulation results show that increasing the ratio of discontinuous to continuous phase velocity increases the bubble size. Using flow-3D software, Chandorkar and Palit [209] simulated the formation and dynamics of microbubbles in a T-shaped microfluidic device. Their simulation results show that with an increasing flow rate of the discrete phase, the bubble size becomes larger. 

Azarmanesh et al. [210] examined several drop formation regimes as well as the influence of the capillary number and Reynolds number on drop formation.

The impact of contact angle on shape and bubble size in a T-shaped microchannel was investigated using a combination of LBM and the free energy technique in this work.

**Figure 20:**
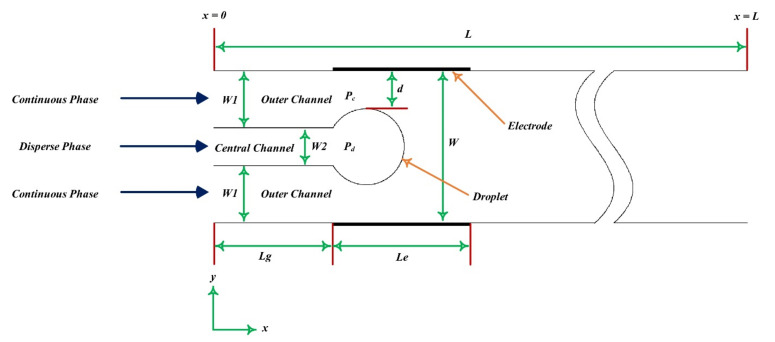
Upstream of a microchannel, diagrams showing the co-flowing of two immiscible fluids [211].

Gong et al. [211] numerically modelled bubble generation in the emulsion process under the influence of the electric field. The influence of an electric field applied to the bubble formation process caused by the mixing of immiscible flows in two-dimensional microchannels with unwept walls was simulated using an intermolecular LBM (Figure 20).

**Figure 21:**
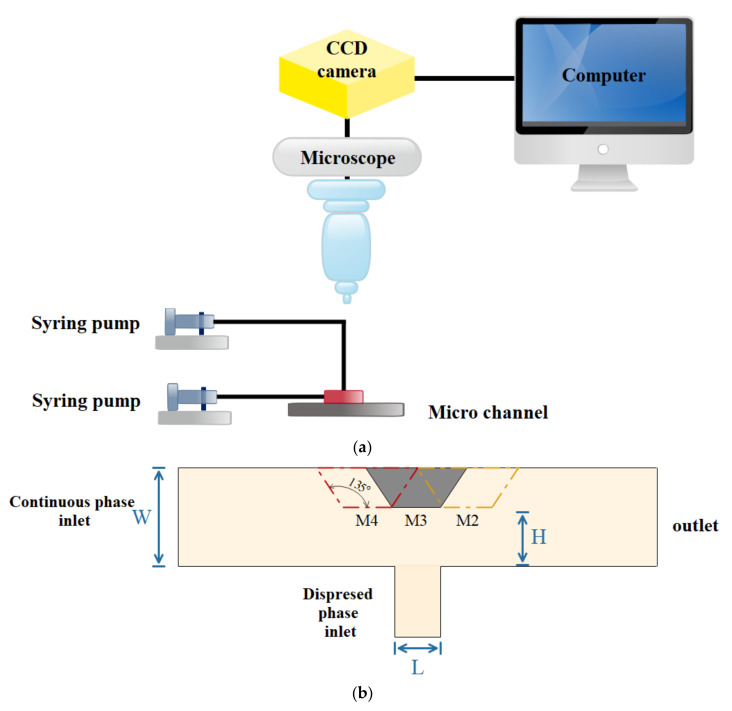
(**a**) Experimental apparatus schematic; (**b**) ordinary and modified T-shaped microchannel schematics [213].

The production of oil droplets in a continuous phase fluid in a microchannel was studied quantitatively by Qiu et al. [212]. The rheology of a continuous fluid was discovered to have a substantial impact on the creation and size of the droplet. Using LBM, Wang [213] reported invaluable results in a novel idea, with a simple change in the T-shaped microchannel (Figure 21). In order to better influence the size and frequency of his drop formation, he used a Venturi-shaped microchannel instead of a conventional T-shaped microchannel. In order to better control the size and frequency of his drop formation, he used a Venturi-shaped microchannel instead of a conventional T-shaped microchannel. The results clearly showed that in the low velocity ratio between phases (velocity between continuous and discontinuous phases), compared to the T-shaped microchannel, in the Venturi-shaped microchannel, smaller high-quality droplets formed. The droplet production in a T-shaped microchannel is based on the equilibrium between the shear force exerted by the continuous phase on the detached phase and the additional Laplace pressure created by the surface tension force. Therefore, changing and manipulating the shear force applied to the discrete separator during the first stage of drop formation can have a significant effect on the drop size and the frequency of its formation. He stated that generally a larger shear force is needed to produce smaller droplets. Moreover, smaller drops provide a larger surface area to increase the mass transfer rate. The results showed that multi-phase flow in micro-devices is highly sensitive to small changes in terms such as channel geometry.

**Figure 22:**
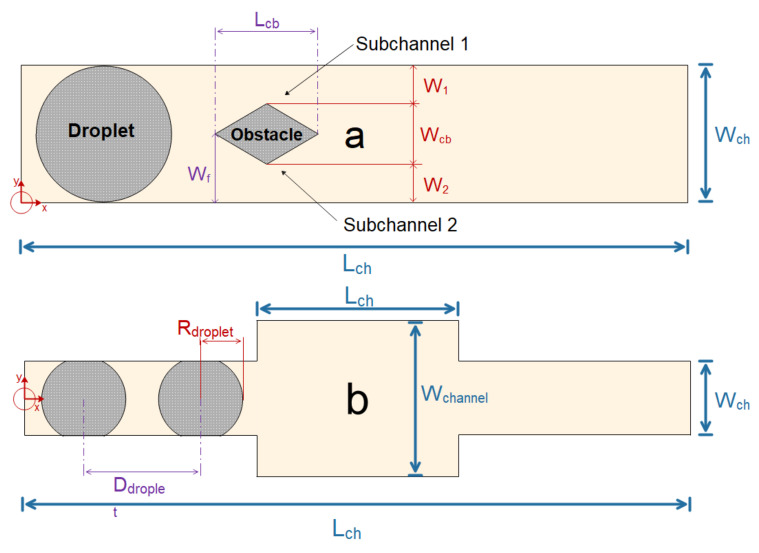
Configuration used for simulation of droplet deformation in a microchannel: (**a**) droplet breakup; (**b**) droplet merging [215].

Garstecki et al. [214] investigated the fundamental physics of droplet generation in T-shaped microchannels in an experimental setting. The pressure differential in the direction of the droplet narrowing affects droplet formation at low capillary numbers (this is called the squeezing effect). The viscosity is the controlling factor in instances when the capillary number is large, and the capillary instability wave induces drop formation. As shown in Figure 22, Lee et al. [215] simulated droplet breakup in the microchannel using Fluent software. The investigated geometry is a microchannel with obstacles in different shapes such as rectangular, rhombic, and circular and drops are splashed by the collision. In this work, the influence of the obstacle geometry on the droplet breakup and the size of the splashed droplets are investigated. A T-shaped microdroplet was investigated by Conchouso et al. [216]. They used the fuzzy field method available in the COMSOL software to track the interface. The purpose of this study was to investigate the effect of channel geometry on particle size and generating smaller particles. Afkhami et al. [217] developed a numerical method to simulate droplet deformation in a microchannel. They used the VOF-HF method for interface tracking and surface tension calculation. In addition to the effect of continuous phase velocity, they investigated the viscosity and surface tension of the discontinuous phase on microdroplet geometry along the channel path.

### 2.3. Combustion in Microchannels

**Figure 23:**
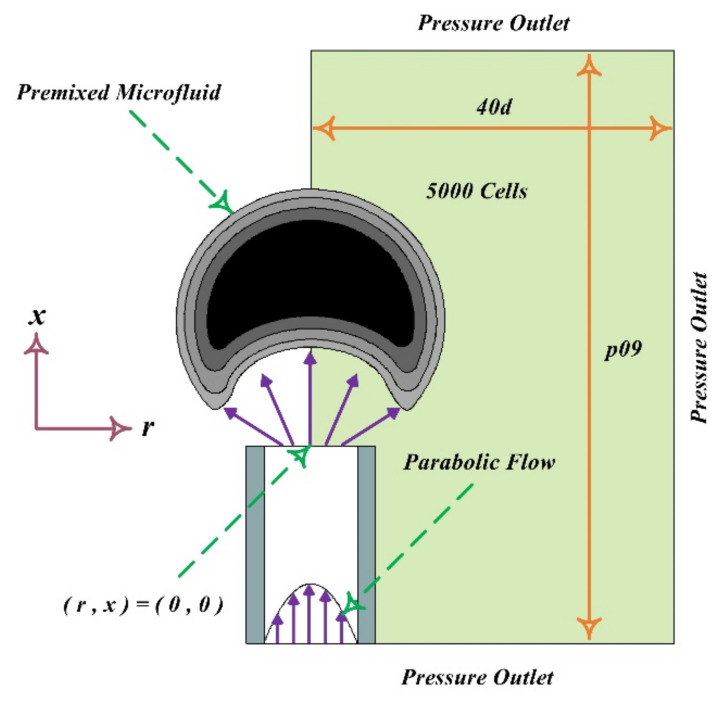
Computational domain [224].

**Figure 24:**
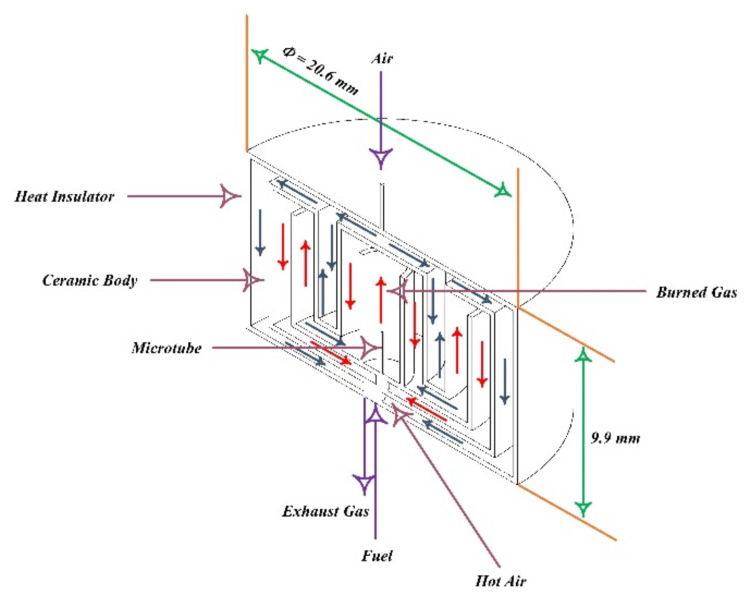
Conceptual design of a heat-recirculated combustor for micropower generation [224].

Combustion is a chemical reaction in which the fuel is oxidized, and large amounts of energy are released. Air is the most common oxidizer used in combustion processes. With the development of science and the increasing need for energy, there is a growing demand for small-scale combustion devices (due to their high energy density). Considering this, it is essential to study the principles of combustion in the microscale and mesoscale. However, due to the high rate of heat dissipation of the walls due to the high surface-to-volume ratio at this scale, thermal management is needed to create a stable flame [218,219,220,221]. Norton and Vlachos investigated the flame stability of microchannels for stoichiometric mixtures of methane–air [222] and propane–air [223] using numerical methods. They looked at how factors including wall thermal conductivity, convection heat transfer coefficient, and fuel–air mixture intake velocity affected flame stability. They also discovered that in such dimensions, the flame extinguishes owing to two primary thermal and kinetic processes. For this form of combustion, the aforementioned processes have a significant impact on the high and low flame velocity. A computational fluid dynamics research was used to explore the combustion parameters and microflame stability characteristics for methane–air premixed combustion. The impacts of microburner size, material thermal conductivity, wall thickness, external heat losses, and operating circumstances on combustion and flame stability parameters were studied using a two-dimensional model. Lee and Kwon [224] investigated the structure and mechanism of the premixed methane–air flame stability in micro dimensions for power generation applications (Figure 23 and Figure 24). Using dimensional analysis, they showed that the ratio of heat dissipation through the wall to the total heat generated by the combustion increases with decreasing dimensions of the combustion chamber. If the aforementioned rate is too high, self-propagating combustion becomes impossible and can lead to thermal extinction for microscale combustion.

**Figure 25:**
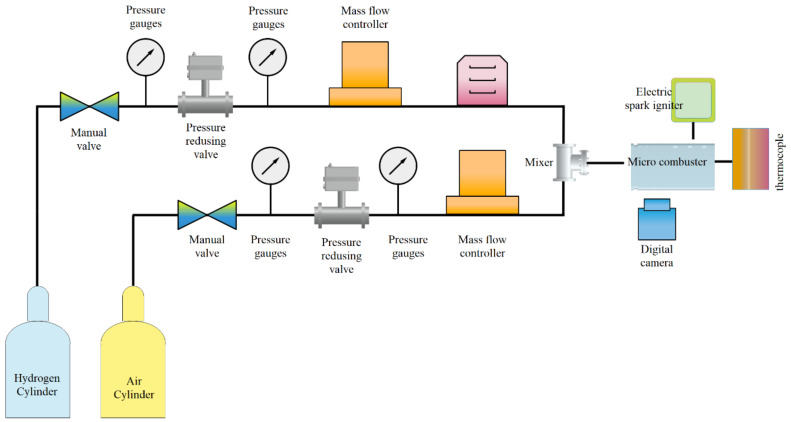
Schematic diagram of the experimental system [231].

By introducing hydrogen to the methane–air mixture in the micro-combustion chamber, Zarvandi et al. [225] examined two-dimensional heat released from the reaction to the wall as well as the influence of heat transmission to the outside wall. They looked at elements including conduction, convection, and radiation heat transfer coefficients that impact external wall temperature distribution and found that when convection and radiation heat transfer coefficients increased, the temperature of the exterior wall fell. Fan et al. [226] looked at how heat transmission, flow field, and flame stabilization interact in a micro-combustion chamber with an obstruction. The quartz combustor has a higher blow-off limit than the stainless steel and SiC combustor, according to numerical simulation findings. Using direct numerical simulation (DNS), Pisa and his colleagues examined the combustion of a dilute hydrogen–air mixture (with an equivalence ratio of 0.5) into heated microchannels, two-dimensionally [227,228] and three-dimensionally [229]. In this study, they reported open axisymmetric flames, extinction, repetitive extinction/ignition, steady symmetric and axisymmetric flames structure, as well as transition regimes such as oscillatory ignition/extinction, but no items were provided on the specifics or causes of these regimes. Premixed methane–air combustion with excessive hydrogen was simulated by Tang et al. [230]. The results of this simulation show that the excess hydrogen has a significant effect on the acceleration of methane reaction rate and flame stability. As shown in Figure 25, the effect of mass flow rate and equivalence ratio on the premixed hydrogen–air flame characteristics in a micro-combustor with an obstacle were examined by Wan et al. [231], experimentally and numerically. The temperature of the flame, its combustion efficiency, and the temperature of the exhaust gas were all investigated. The results demonstrate that as the equivalency ratio is increased from 0.4 to 0.6, the blow-off limit increases.

**Figure 26:**
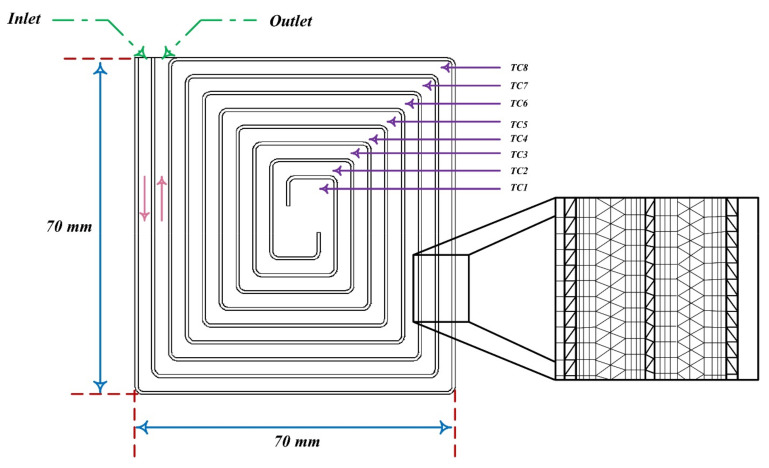
Geometry and grid structure of the modeled Swiss-roll combustor [233].

The effect of bluff body shape in a micro-combustion chamber on the flame stability of an air–hydrogen mixture was studied by Bagheri et al. [232]. In this study, combustion efficiency, wall temperature, exhaust gas temperature, and flame temperature have been examined by placing bluff bodies with different shapes within the channel, which narrows the inlet cross-section of the air–hydrogen mixture. The turbulent premixed hydrogen–air flow within the combustion chamber, shown in Figure 26, was numerically simulated by Kuo and Ronney [233]. The Reynolds number of 500 was defined as the threshold of the turbulent flow regime. He also observed that the contribution of radiation heat transfer is greater than the convection mode of heat transfer.

## 3. Applications of Microscale 

### 3.1. Micromixers

**Figure 27:**
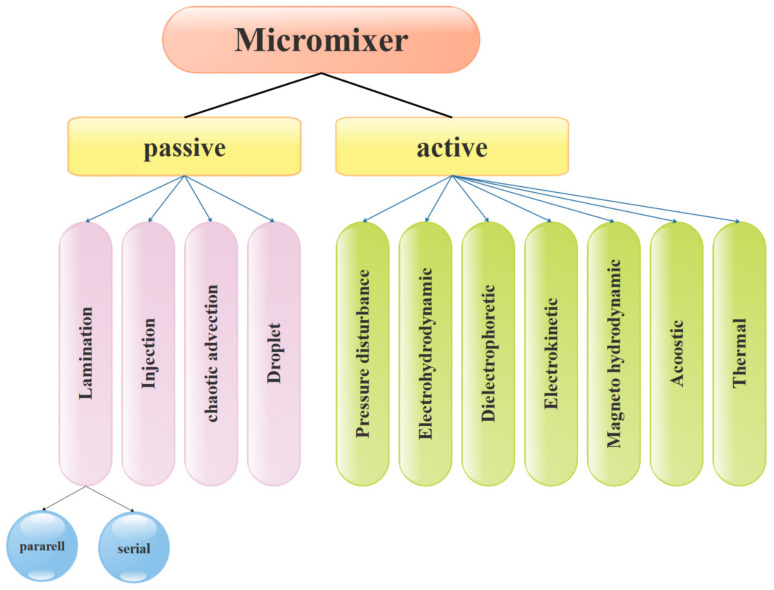
Classification of micromixers [238].

Increasing applications of microfluidic technology are currently visible in analytical chemistry and chemical production. The technology of microfluidic systems has covered a wide range of fundamental studies and actual applications in industry and laboratories. In the study of micromixers, it is necessary to first be acquainted with the concept of mixing. Mixing is called the process of changing a heterogeneous system into a homogeneous system. Mixing operations usually involve mixing two volumes of fluids, chemical reaction, heat transfer, mass transfer, or multi-phase combination (suspension and suspension) to reduce inhomogeneity in the industry. As a result, secondary effects such as response and property change occur. Mixing is also a technique utilized in most microfluidic devices for medical diagnostics, chemical manufacturing, drug discovery, and other applications [234,235,236]. The three traditional mixing methods are macromixing, mesomixing, and micromixing. Macromixing governs the largest scale of fluid motion and happens in a turbulent flow regime. As a result, the two fluids’ contact area grows, and the mixing length shrinks. The smallest scale of fluid motion in the macromixing is the eddy size. Micromixing occurs at the molecular dimension. The mixing mechanism for the transfer of mass, heat, as well as the chemical reaction at the interface where these phenomena occur, is micromixing. Mesomixing is a scale between macromixing and micromixing, which is the combination of the two levels [237]. Micromixers are divided into two types, as indicated in Figure 27: active and passive. Passive micromixers do not require external pumping power, but active micromixers must [238].

**Figure 28:**
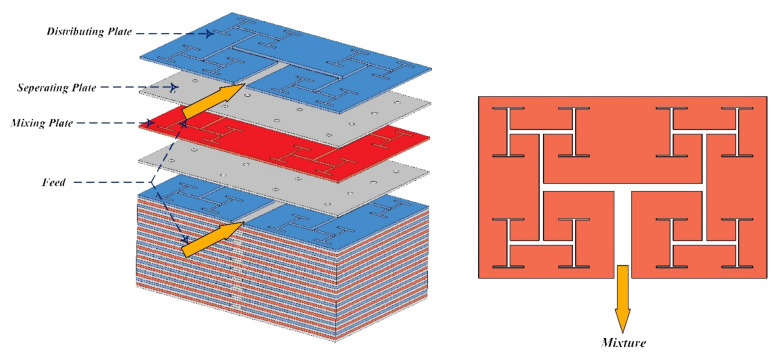
The construct tree-shaped configuration for a micromixer [241].

**Figure 29:**
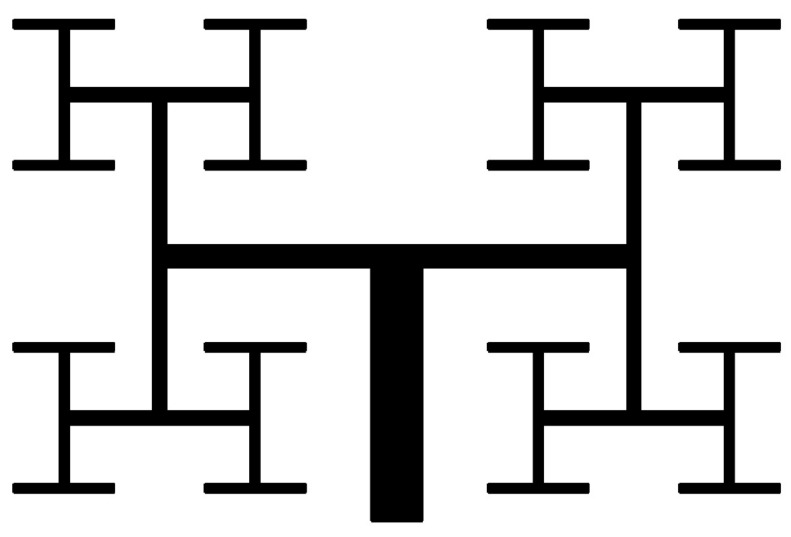
A typical tree-shaped configuration filling in a rectangle plane [241].

To enhance mixing efficiency, active micromixers rely on external energy, such as pressure difference, temperature, magnetic, and electric fields. These micromixers are capable of mixing a wide range of fluids. Since passive micromixers do not require complex systems to increase mixing efficiency, the development of active micromixers today is not very welcomed. In passive micromixers, turbulent flow produces using obstacles, and the main flow turns into smaller flows. Channel deformation is another method by which the two-fluid interface area is increased and results in increased mixing efficiency [238,239]. Thus, compared to active micromixers, the investigation of the flow in passive micromixer channels is complex, and the evaluation of the effect of different geometrical parameters on mixing performance is important. Passive micromixers try to manipulate the interface between the mixing fluids and increase the mixing rate using the special geometry of these devices, which include elbows and curved channels. Since no external energy is used by this type of micromixers, the mixing length and their size are greater than active micromixers. Due to the dominance of the laminar flow in these micromixers, the mixing is incorporated with the molecular diffusion mechanism and disordered and the bulk irregular motion. These micromixers increase the molecular diffusion rate by increasing the interface area and by decreasing the penetration path. These types of micromixers are classified into five classes of multi-lamination, split-and-recombine, chaotic advection, injection, and droplet. Kanaris et al. [239] investigated six microchannel designs including T-shaped, C-shaped, and L-shaped micromixers, simple orthogonal helix micromixer, single-plane curved spiral micromixer, and two-plane curved spiral micromixer, numerically. They found that the L-shaped micromixer performed slightly better than the C-shaped micromixer and the curved spiral geometry on two parallel plates. Afzal and Kim [240] presented a passive micromixer with a converging–diverging channel with sinusoidal changes. They observed that in the Reynolds numbers of 10–70, the secondary flow was stronger and the mixing performance improved. As shown in Figure 28 and Figure 29, mixing in a tree-shaped configuration was studied, numerically and experimentally, by Wang et al. [241]. They concluded that increasing Reynolds number and number of branches improved mixing performance.

**Figure 30:**
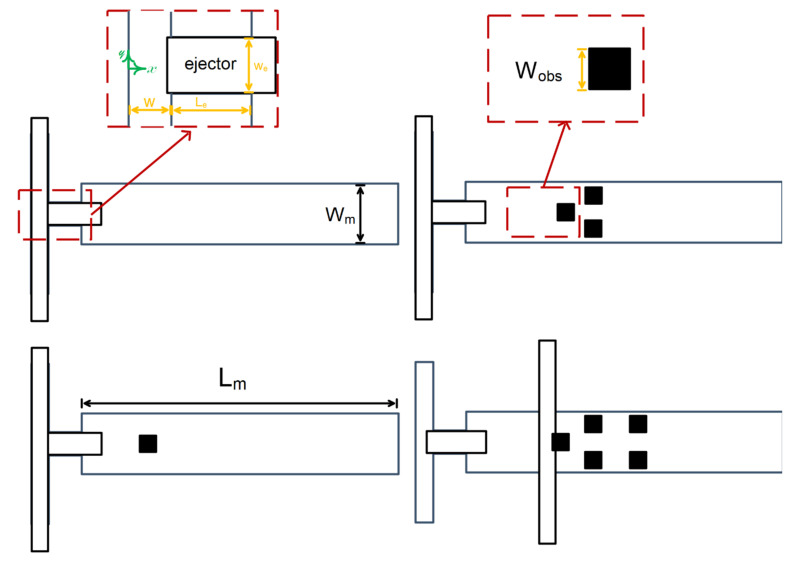
Schematic representation of the mixers studied [245].

Solehati et al. [242] have proposed a wavy micromixer design. Compared to straight micromixers, oscillating secondary flow and consequently irregular flow regime improves the mixing in this micromixer. Mixing in the aforementioned micromixer under a pressure difference was studied semi-analytically by Chen et al. [243]. Their solution was then improved by Song et al. [244] by proposing a fully-analytical method. The results of the method used to solve the mass transfer equation under pressure gradient were compared with the literature data. Subsequently, they were provided a detailed analysis of the mixing in Y-shaped micromixers, taking into account the Helmholtz–Smoluchowski velocity at the surface. Miranda et al. [245] investigated the mixing in the micromixer shown in Figure 30 with pulsatile input flows using computational fluid dynamics, two-dimensionally. The best mixing was when the velocity phase difference between the two inlet flows was 180° and the number of square obstacles in the middle of the outlet channel was greater.

**Figure 31:**
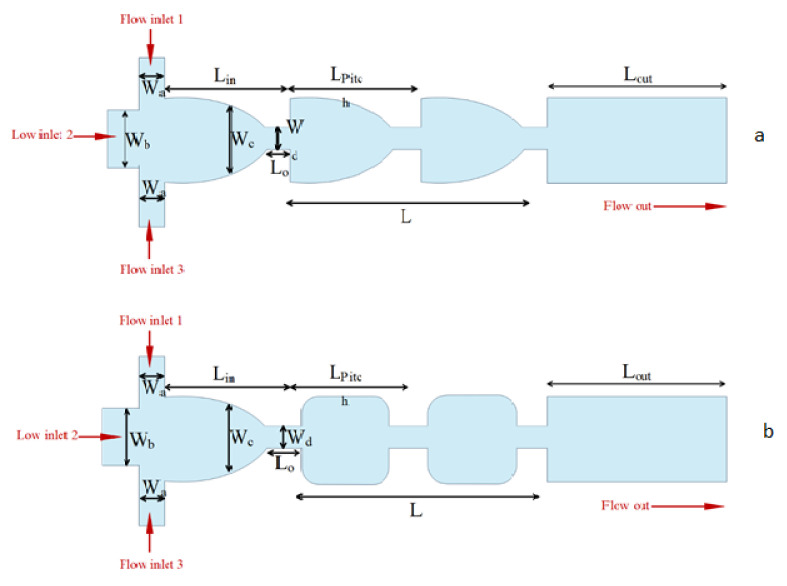
(**a**) Hexagonal chamber (H) and (**b**) round corner rectangular chamber design of planar micromixer (RCR) [249].

Mixing of water and blood, respectively, as a Newtonian and as a non-Newtonian fluid in T-shape and sinusoidal serpentine micromixers were compared by Afzal and Kim [246]. They found that for any values of flow rate, the serpentine micromixer had better mixing. Moreover, for both Newtonian and non-Newtonian fluids, the mixing decreases and then increases with increasing flow rate. The results also showed that at any fixed value of flow rate, the pumping power required in the non-Newtonian fluid flow was much higher than the Newtonian fluid flow. In the Reynolds numbers of 0.1–60, Alam et al. [247] quantitatively studied the mixing of water and ethanol in a curved micromixer, as well as the influence of different forms of obstacles. They observed that the micromixers with circular and hexagonal obstacles had the same mixing degree and the micromixers with rhombic obstacles had a lower mixing rate than the other micromixers. Bahrami et al. [248] applied a magnet to boost mixing efficiency in the microchannel. They revealed that there is an optimum point for the mixing index as the number of magnets goes up from one to three magnets. Chery et al. [249] experimentally and numerically studied mixing within plane micromixers with a small mixing length (Figure 31). Water and ethanol flow was solved in two mixer configurations along with four obstacle shapes in five Reynolds numbers in the range of 0.1–40 using three-dimensional Navier–Stokes equations. From these 40 cases, the optimum micromixer, which has mixing degrees of 0.89 and 0.99 for Reynolds numbers of 0.1 and 40, respectively, in a mixing length of 1.18 mm, was selected. Then, the optimized micromixer was constructed, and it was found that the numerical and experimental results were in good agreement.

**Figure 32:**
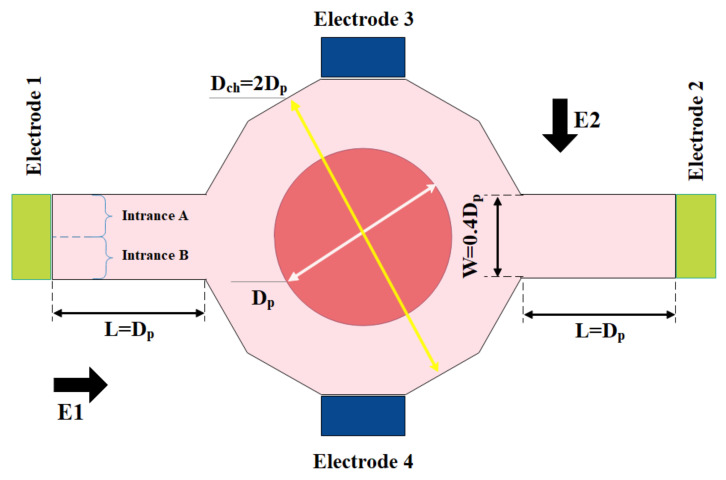
Diagram of the proposed micromixer, showing two microchannels and one suspended particle inside the microchamber [250].

Daghighi and Li [250] presented a novel design of a micromixer based on electrokinetic as in Figure 32. The mixer consists of a cylindrical chamber that is connected to two microchannels at the inlet and outlet of the channel, with suspended, fully conductive, circular particles. By applying an external electric field, vortices are created around the conductive particle and increase the mixing percentage. The final position of the particle and the vortex strength depend strongly on the applied electric field. They found that applying an electric field at a 45-degree angle increased the mixing percentage. Moreover, by applying a stronger electric field, the mixing time is reduced.

A T-shape micromixer with pulsatile inlet velocity was examined experimentally and numerically by Ma et al. [251]. Optimization was performed. Minakov et al. [252] investigated the slip boundary condition in a T-shaped micromixer to establish mathematical modeling. At the channel output, the Neumann boundary condition is imposed (the derivatives in the vertical direction of the output are zero). They stated that using a hydrophobic coating increases the slip length within the microchannel and thus reduce the pressure drop. In low Reynolds numbers, considering and neglecting the slip boundary condition has no significant effect on the flow patterns. The slip condition is given by u = b (∂u/∂n) where b is the slip length (b from 1 ϻm–70 ϻm). As the slip length increases, the two S-shaped vertexes become one vertex, which results in increased mixing efficiency and low pressure drop. Hashim et al. [253] simulated a microfluidic with three inlets using COMSOL software. They studied how mixing of two liquids, concentration distribution, and flow pattern. The presence of obstacles in pressure-driven flow within Y-shape microchannels was experimentally and numerically studied by Wang and Hu [254]. By studying the geometry, location, and number of obstacles in different Reynolds numbers, an optimal design was proposed to increase mixing efficiency. They also showed that the presence of an obstacle increased the convection and improved mixing.

### 3.2. Microturbines

**Figure 33:**
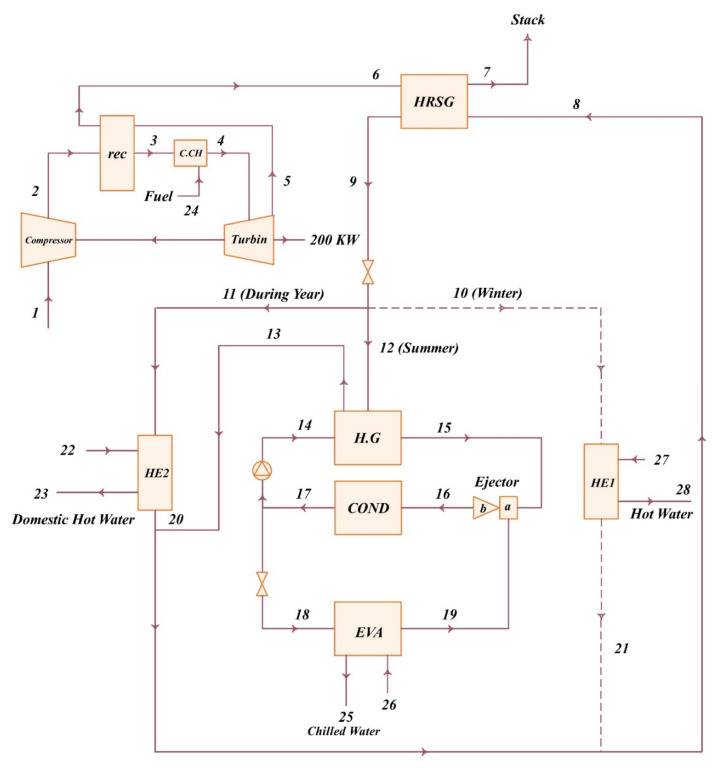
Schematic of the microturbine cogeneration system with the steam ejector refrigeration system [255].

Microturbines are one of the newest types of heat turbines used to generate electricity and heat. They are simple and small gas turbines with a power output of about 30 to 500 kW. The electrical efficiency of the microturbines, as shown in Figure 33, decreases with the increasing ambient temperature. The below equation shows how the efficiency of the microturbine changes with the temperature [255]:

(4) $$\eta_{T}=30.8-\left(0.12\times T_{amb}\right)$$

where $T_{amb}$ is the ambient temperature in celsius.

Microturbines can be used in a wide range of applications related to electrical and thermal power generation. Compared to other energy generation equipment and technologies, these devices have features such as high efficiency (when using electricity and heat at the same time), least moving elements, low weight, simultaneous production of power and heat, very low environmental pollution, low maintenance cost, usability of different fuels, low cost of electricity generation, and so on. The use of microturbines in combined electrical and thermal power generation systems can double their overall efficiency compared to those used only for generating electricity. Due to favorable characteristics of microturbines, their usage in different applications of electric and thermal power generation in industrial, commercial, and etc., is increasing day by day. Ehyaei and Bahadori [256] examined the performance of a microturbine designed to meet the electrical, heating, and cooling needs of a building. In this study, they calculated the power requirements of the system for three different cities in Iran overnight. In another study, they examined the same system from an economic point of view [257]. Ameli et al. [258] analyzed a gas microturbine-based cogeneration system. In this simulation, they calculated the heating, cooling, and electrical needs of a building throughout the year and designed a gas-based microturbine cogeneration system.

### 3.3. Micropumps

Chatterjee and Amiroudine [259] used LBM to look into the thermodynamic characteristics of MHD fluid flow in a micro pump and provided a non-isothermal method. The slip velocity boundary condition was not included in the computations, and the flow was incompressible, according to the equations and simulation findings. Using LBM, in order to minimize the entropy generation, Khozeymeh-Nezhad and Niazmand [260] investigated the influence of the geometrical and functional parameters of a viscous micropump and determined the optimal range. Many studies have been conducted on the factors that influence the performance of MHD flows in micropumps. The Lorentz force generated by the magnetic field pushes the electrically conductive fluid through the channel in this type of pump. The performance of MHD micropumps has been investigated by Ho [261] assuming a steady, incompressible, fully developed laminar flow. The findings of their study were consistent with those of experimental investigations.

Kiyasatfar et al. [262] examined the fluid flow from the MHD micropump in a rectangular cross-section channel. The effects of magnetic flux density, channel diameters, and electric current on velocity profiles and temperature distribution were the focus of their study. Their findings revealed that as the Hartmann number rises, fluid velocity and temperature rise as well. Ito et al. [263] studied the effect of channel dimensions on the flow patterns in a MHD micropump.

## 4. Challenges, Suggestions and Conclusions

According to the assessment of previous studies and considering the human needs for fabricating various devices with small sizes, the study of fluid flow in micro and nanoscales is of great importance. Since the behavior of fluid flow in these dimensions has special properties, the study of fluid mechanics in micro and nanoscales seem necessary. Therefore, attention to this area has increased and a lot of studies have been conducted in recent years. Thus, the present study aimed to review previous studies of fluid flows in microstructures to provide a comprehensive source for future studies by providing a summary of previous articles in order to pave the way for the authors. First, an introduction to microfluidics was presented. Then, modeling methods were evaluated, and finally, the results were assessed. At the end of the article, the existing challenges in this field are presented and suggestions are given for future studies.

According to the examination of the previous papers, the experimental results demonstrated that the pressure drop profile for two-phase flows is significantly different from the one for single-phase flows in a very narrow microchannel. For similar microchannels, the resistance to single-phase flow remains constant after the initial increase due to fluid acceleration. However, for two-phase flows, the resistance increases sharply when the interface of the two-phase enters a microchannel with a diameter smaller than a certain size. Whenever the microchannel has the optimal value proposed in the studies, the microchannel imposes a force on the interface and the resistance to the two-phase flows suddenly enhances.

It is also found that immiscible two-phase flow in porous media in various fields, such as oil refinery, carbon dioxide separation, groundwater reclamation, water management in fuel cells, electronic chips, compact heat exchangers, biotechnology, and micro-drug delivery bubbles, are of particular importance. In many cases, resistance to two-phase flows in porous media is used as a criterion for detecting the convection and transport processes and interaction between microchannels and fluids in closed enclosures. Microchannel resistance to fluids is usually assessed by measuring the pressure drop across fluids passing through the channel. The fluid flow is controlled by the structure of the throat (such as size and shape of the throat), throat surface moisture, fluid properties, such as viscosity and surface tension, fluid velocity, and the interface between two fluids.

Flow in microchannels has been extensively studied over the past two decades for efficient and faster cooling of high-density electronic devices. The high heat transfer coefficient in microchannels can significantly reduce the size of heat exchangers. Other advantages of microchannels include their low weight, low volume, and low material used. Reducing the diameter of microchannels in most compact heat exchangers enhances the heat transfer coefficient due to the larger surface area per unit volume. Microchannels have a wide range of practical applications in many specialized fields, including bioengineering and microstructure flow systems, micropumps, and thermal microtubes. For example, the low density and weight of microchannels improve the automotive industry. Small heat exchangers for microchannels have now been employed instead of circular tubes in car condensers and heat exchangers with hydraulic diameters of about 1 mm. Recently, microchannels have been successfully used in car air conditioning systems, fuel cells, and microelectronics. The main challenge of microchannels is the difficulty of fabricating and filtering high-grade working fluid to flow through the channels. Pressure drops and the required pumping power are the challenges of microchannels.

Nanotechnology and microtechnology have now changed the prevention, diagnosis, and treatment of diseases. In any case, the capability of nanotechnology and microtechnology is more than other options. Microfluidic technology has reached a point of maturity and is a reliable platform for new medical applications. Many microtechnologies have entered the market, facilitating the diagnosis phase and enabling continuous monitoring of items, such as metabolism and vital signs. Pregnancy and parenting tests are readily available. Conversely, there is a need to expand the applications of POC for cost-effective diagnoses of infectious diseases (Ebola, influenza, and malaria), especially for underdeveloped countries. Traditional technologies are not enough to solve global health problems, so the need for cost-effective and portable POC technologies is another important factor in the application of micro and nanotechnologies. Integrating simple and creative ideas can produce q2. Micro- and nano-devices are now available, such as nano-sensors, antibodies, NPs, quantum points, etc. The main concern is the relatively high price of complete antibodies, which are the most expensive material in POCs. The use of antibody components is considered as a technique to reduce the price of POCs. The availability of rapid sensors and biosensors has contributed to the development of efficient diagnostic and monitoring devices.

Many microtechnologies will be developed in laboratories in the coming years. Microelectroporation and organ-on-a-chip applications are rapidly gaining acceptance in laboratories around the world. Biotechnology companies and pharmaceutical and biology companies are expected to be the main customers of lab-on-a-chip devices. Organ-on-a-chip platforms will be used in the future for the initial phases of drug production by large laboratories and pharmacies. Other aspects of this technique are currently being explored.

Nanotechnology in the field of diagnosis and treatment is transitioning from idea to action. The need to deliver the drug with high accuracy to specific targets has made the delivery devices smaller and more stable. As the scales become smaller, the surface-to-volume ratio is enhanced. Nanoparticle-based therapeutic applications have higher selectivity and greater efficacy than conventional ones. These improvements mean more efficiency for patients with fewer side effects. NPs, for example, are well-suited for delivering many drugs and nucleic acids because more penetration to tissues is possible.

The early stages of the development of nano-based research have been very good but ensuring the safety and efficacy of these products needs to be transferred to clinical trials as a next step. Important barriers to transitional research generally apply to nanomedicine as well. The following is a brief overview of several technical and non-technical issues related to nanotechnology, including budget, preparation aspects, and regulatory issues.

Many related issues need to be addressed before nanoproducts can be widely used. Before these drugs reach patients, safety concerns and strict regulations must be considered. The budget required for the transition from research to clinical trials should also be considered.

Cancer drugs are undoubtedly the forerunners of nanotechnology drugs in medicine, although NPs have been shown to have other uses, such as drugs and genes delivery to heart tissue. Designing antibacterial surfaces using NPs has opened up a new way to combat infections without the use of antibiotics and related drug resistance. Nano-production is expected to have unprecedented applications in nanotechnological diagnosis and treatment by using nucleic acids or cutting and pasting genetic codes. Nanoscale strategies use membrane curvature to combat bacterial resistance.

Our ability to imitate creation in combating disease provides more strategies for researchers. Our creation has inspired us to design and fabricate materials with micro and nanostructures to transfer new physical and chemical properties to surfaces and devices. Nature can teach us how to achieve surface adhesion underwater, how to move fluids through complex channels, and enhance blood circulation time.

Despite such problems, the development of nanomedicines is very good in all areas of treatment. This improvement is particularly noticeable in the field of cancer, where the number of clinical trials has enhanced ten-fold in the last decade. In addition, many nano-based platforms for reformulating and presenting new molecular identities are improving current drugs.

There are many resources to fund and support emerging research and discoveries related to nanotechnology. The transition from pre-clinical to clinical research requires large investments that are not usually supported by traditional governments and non-profit organizations. These conditions create a large gap between the initial stage of discovery and the later stages of clinical trials (usually called the Death Valley stage). Many federal budget sources have realized this problem and have devoted part of their non-organizational budget to this field.

The lack of clear law and related procedures has led to many security and risk efforts, which increases costs and disrupts production stability. It should be noted that several researchers and start-ups have received rapid legal approvals (less than 6 months) for their nanostructured medical devices (usually orthopedic), and these approvals were given when they used nanoscale surfaces. New materials were not used to improve tissue growth, prevent infection, and reduce swelling. Such approaches have been used to rapidly commercialize medical devices.

One of the most popular subfields of artificial intelligence is machine learning, which is widely considered these days. Therefore, it is suggested the use of this branch in micro- and nano-devices to provide an optimal model. Machine learning can be considered by researchers in future studies.

By evaluating the different methods of production of microreactors, it can be concluded that the design of microreactors is the most important step in their production. In other words, the selection of an unsuitable design will cause errors in the results of the designed reactor due to the major differences in the design and fabrication of microreactors and conventional-scale ones. Meanwhile, microreactors made of glass are used in many reactions due to their low cost and high flexibility in different operating conditions. On the other hand, in special operating conditions such as high temperatures, metal, and ceramic are the only available options for fabricating microreactors.

Biological imitation involves copying creation in all its details. Although all problems cannot be solved by imitating nature alone, inspiration from nature can help solve many problems. A general idea in nature can be indigenous and even improved for a specific case. Consider that every alive creator around us has overcome many difficulties during evolution, and this puts a world of new ideas in front of us. Looking at nature, we must understand the problem well and be able to use it. This requires a multi-purpose team.

The integration of micro and nanotechnology can revolutionize the diagnosis and treatment of infectious and chronic diseases and provide us with new platforms for the study of tissues, cells, and molecules.

## Figures and Tables

**Table 1:** Some research on multi-phase flow in micro-equipment.

Research Method	Methods and Type of Paper	Device	Ref
Numerical CFD simulation	Multi-phase flow (VOF)	Microchannel heat exchangers	Panda et al. [53]
Numerical 3D CFD simulations	Multi-phase flow (VOF)	Straight and spiral microchannel	Chatterjee et al. [54]
Numerical Lattice Boltzmann method	Multi-phase lattice Boltzmann method	Impingement on a rigid square obstacle in a microchannel	Bakhshan et al. [55]
Numerical 3D CFD simulations	Multi-phase flow (VOF)	Falling film microchannel	Chen et al. [56]
Experimental	Multi-phase blood flow	Microchannel	Lima et al. [57]
Numerical methods Experimental Application	Review	Microfluidic systems	Sattari et al. [58]
Experimental	Multi-phase flow Liquid/liquid microfluidic flows	Coaxial micro-device	Dinh and Cubaud [59]
Numerical 3D CFD simulations	Multi-phase transesterification reaction Multi-phase flow (VOF)	Microchannel reactor	Laziz et al. [60]
Mixing and mass transfer Two-phase micro-flow Bubble/droplet formation	Review	Two-phase flow and mass transfer in microchannels	Yao et al. [48]
Numerical simulations Droplet’s formation behavior	OpenFOAM InterFOAM solver using the VOF	Micro-T-junction channel	Guimarães et al. [61]

**Table 2:** A classification of channels based on hydraulic diameter.

	Conventional Channels
$200\;\mu m>D_{h}>3\;mm$	Mini channels
$10\;\mu m>D_{h}>200\;\mu m$	Microchannel
$0.1\;\mu m>D_{h}>10\;\mu m$	Transition channels
$D_{h}<0.1\;\mu m$	Molecular nanochannels

**Table 3:** Channel dimensions for types of gas flow at atmospheric pressure [62].

Channel Dimensions μm				
Continuum Flow	Slip Flow	Transition Flow	Free Molecular Flow	Gas
>67	0.67–67	0.0067–0.67	>0.0067	Air
>194	1.94–194	0.0194–1.94	>0.0194	Helium
>123	1.23–123	0.0123–1.23	>0.0123	Hydrogen
